# Sorbitol as a Polar Pharmacological Modifier to Enhance the Hydrophilicity of ^99m^Tc-Tricarbonyl-Based Radiopharmaceuticals

**DOI:** 10.3390/molecules25112680

**Published:** 2020-06-09

**Authors:** Carolina Giammei, Theresa Balber, Katarina Benčurová, Jens Cardinale, Neydher Berroterán-Infante, Marie Brandt, Nedra Jouini, Marcus Hacker, Markus Mitterhauser, Thomas L. Mindt

**Affiliations:** 1 Ludwig Boltzmann Institute Applied Diagnostics, General Hospital of Vienna, c/o Sekretariat Nuklearmedizin, Währiger Gürtel 18-20, 1090 Vienna, Austria; carolina.giammei@lbiad.lbg.ac.at (C.G.); theresa.balber@lbiad.lbg.ac.at (T.B.); katarina.bencurova@lbiad.lbg.ac.at (K.B.); jens.cardinale@lbiad.lbg.ac.at (J.C.); marie.brandt@lbiad.lbg.ac.at (M.B.); nedra.jouini@lbiad.lbg.ac.at (N.J.); markus.mitterhauser@meduniwien.ac.at (M.M.); 2 Department of Inorganic Chemistry, Faculty of Chemistry, University of Vienna, Währinger Strasse 42, 1090 Vienna, Austria; neydher.berroteraninfante@meduniwien.ac.at; 3 Department of Biomedical Imaging and Image-Guided Therapy, Division of Nuclear Medicine, Medical University of Vienna, Währinger Gürtel 18-20, 1090 Vienna, Austria; marcus.hacker@meduniwien.ac.at

**Keywords:** ^99m^Tc-tricarbonyl, bombesin, click-to-chelate, glycation, sorbitol, pharmacological modifier, hydrophilicity, radiolabeled peptides, SPECT

## Abstract

The organometallic technetium-99m tricarbonyl core, [^99m^Tc][Tc(CO)_3_(H_2_O)_3_]^+^, is a versatile precursor for the development of radiotracers for single photon emission computed tomography (SPECT). A drawback of the ^99m^Tc-tricarbonyl core is its lipophilicity, which can influence the pharmacokinetic properties of the SPECT imaging probe. Addition of polar pharmacological modifiers to ^99m^Tc-tricarbonyl conjugates holds the promise to counteract this effect and provide tumor-targeting radiopharmaceuticals with improved hydrophilicities, e.g., resulting in a favorable fast renal excretion in vivo. We applied the “Click-to-Chelate” strategy for the assembly of a novel ^99m^Tc-tricarbonyl labeled conjugate made of the tumor-targeting, modified bombesin binding sequence [Nle^14^]BBN(7–14) and the carbohydrate sorbitol as a polar modifier. The ^99m^Tc-radiopeptide was evaluated in vitro with PC-3 cells and in Fox-1^nu^ mice bearing PC-3 xenografts including a direct comparison with a reference conjugate lacking the sorbitol moiety. The glycated ^99m^Tc-tricarbonyl peptide conjugate exhibited an increased hydrophilicity as well as a retained affinity toward the Gastrin releasing peptide receptor and cell internalization properties. However, there was no significant difference in vivo in terms of pharmacokinetic properties. In particular, the rate and route of excretion was unaltered in comparison to the more lipophilic reference compound. This could be attributed to the intrinsic properties of the peptide and/or its metabolites. We report a novel glycated (sorbitol-containing) alkyne substrate for the “Click-to-Chelate” methodology, which is potentially of general applicability for the development of ^99m^Tc-tricarbonyl based radiotracers displaying an enhanced hydrophilicity.

## 1. Introduction

Radiolabeled peptides have become an indispensable tool in nuclear medicine for the diagnosis (imaging) and therapy of cancer. In the majority of cases, radioactive metals are used in this context. Application of radiometal complexes for the radiolabeling of peptides has the advantage that the metal is exchangeable and thus, both diagnostic and therapeutic probes become accessible depending on the radionuclide employed. This approach has been termed “theranostics” [1]. Different combinations of radiometals have been described in the literature for theranostic approaches [2], for example the matched pair of the group 7 transition metals technetium-99m (γ-emitter for imaging) and rhenium-186/188 (β^-^-emitter for therapy) [3]. Technetium-99m (^99m^Tc) is considered the workhorse of nuclear medicine among the single photon emitting radionuclides for molecular imaging by SPECT due to its optimal physical properties (t_1/2_ = 6 h, E_γ_ = 140 keV) and broad availability. After being eluted from the generator as pertechnetate, ^99m^Tc(VII)O_4_^−^ must be reduced to a lower oxidation state for the coordination with a chelating system. Among different reported ^99m^Tc precursors, also referred to as ^99m^Tc cores [4], the ^99m^Tc-tricarbonyl core, [^99m^Tc][Tc(CO)_3_(H_2_O)_3_]^+^, is an interesting and promising candidate that can be readily prepared via the reduction of ^99m^Tc(VII) to ^99m^Tc(I) using commercial kits [5]. However, a potential drawback of the organometallic ^99m^Tc-tricarbonyl precursor for radiometallation of (bio)molecules is its increased lipophilicity in comparison to other ^99m^Tc-cores and radiometals, which can impact the pharmacokinetic profile of a tumor-targeting radiotracer. Lipophilic radiolabeled peptides can show hepatic uptake and slow hepatobiliary excretion instead of fast renal elimination, which is a favored characteristic of tumor-targeting imaging probes [6]. To overcome such issues, a number of radiolabeled peptides have been modified synthetically by conjugating polar pharmacological modifiers such as carboxylates [7,8,9], oligo- and polyethylene glycols [10,11], or carbohydrates [12,13].

**Figure 1:**
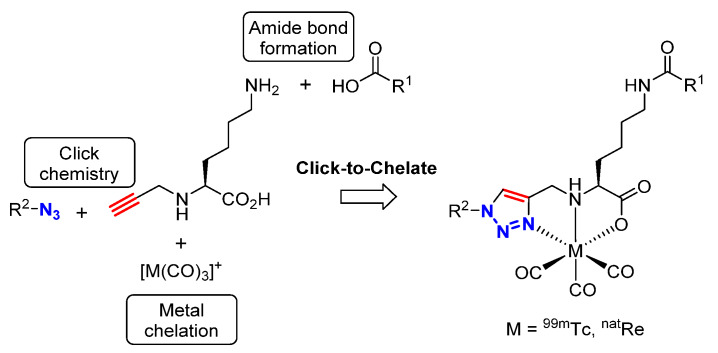
Assembly of ^99m^Tc-labeled conjugates by “Click-to-Chelate”.

An elegant methodology to introduce a chelator for the ^99m^Tc-tricarbonyl core into (bio)molecules is via the “Click-to-Chelate” approach (Figure 1) [6,14]. This strategy includes an application of the Cu(I)-catalyzed azide-alkyne cycloaddition (CuAAC) [15,16] by which an efficient tridentate chelator for the ^99m^Tc-tricarbonyl core is formed and linked to (bio)molecules of interest while other moieties, e.g., carbohydrates, can be conjugated simultaneously [17]. We have previously applied this methodology to the assembly of a peptide conjugate comprising a modified analog of the binding sequence of the tumor-targeting peptide bombesin (BBN), namely [Nle^14^]BBN(7–14), a ^99m^Tc-tricarbonyl complex and the carbohydrate glucuronic acid [18]. BBN is an attractive peptide for targeting the Gastrin-releasing peptide receptor (GRPR), which is overexpressed by a variety of tumors, including prostate and breast cancer [19]. The aim of the study was to enhance the hydrophilicity of the radiolabeled peptide and therefore favoring renal over hepatobiliary excretion. While the hydrophilicity of the glycated peptide conjugate was found substantially increased, we were surprised that it accumulated nevertheless to a higher degree in the spleen and liver in comparison to a more lipophilic BBN reference compound. We assumed that glucuronated, negatively charged metabolites of the radiolabeled peptide can be recognized by organic anion transporting polypeptides (OATPs), which are expressed in these organs [18].

We thus resorted to the use of other carbohydrates as pharmacological modifiers. Reduced acyclic carbohydrates lacking potentially charged functional groups appeared as a promising alternative. For example, sorbitol is used safely as an artificial sweetener in food products. Weinstein et al. have reported that [^18^F]fluorodeoxysorbitol has potential as an infection imaging agent for positron emission tomography (PET) and does not accumulate in the spleen and liver [20]. Also Leamon et al. showed that sorbitol derivatives are promising polar moieties for preventing hepatic clearance of drugs [21]. We thus hypothesized that the sorbitol moiety might present an appropriate innocent pharmacological modifier for our purpose. Herein, we report the “Click-to-Chelate” synthesis, radiolabeling and in vitro and in vivo evaluation of a novel glycated (sorbitol-containing), ^99m^Tc-tricarbonyl labeled [Nle^14^]BBN(7–14) conjugate including a side-by-side comparison with a reference compound lacking the carbohydrate moiety.

## 2. Results and Discussion

### 2.1. Syntheses

**Scheme 1:**
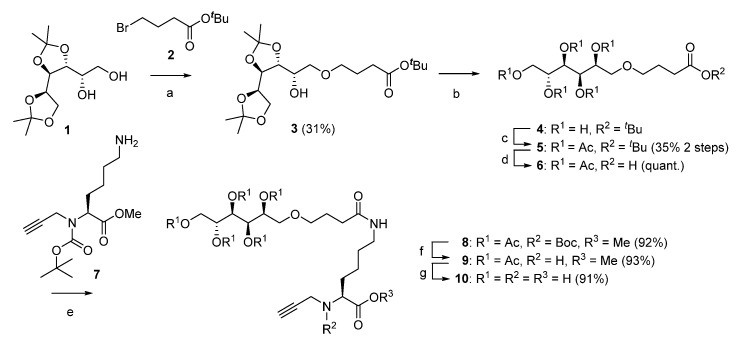
Synthesis of Cu(I)-catalyzed azide-alkyne cycloaddition (CuAAC) alkyne substrate **10**. a) i) Bu_2_SnO, toluene, Dean-Stark, reflux 24 h, ii) tert-butyl 4-bromobutanoate (2), toluene, reflux, 24 h; b) i) AcOH (80%), rt, 18 h, ii) CHCl_3_, rt, 18 h; c) Ac_2_O, pyridine, CH_2_Cl_2_, rt, 24 h; d) CH_2_Cl_2_/TFA (3:1), rt, 18 h; e) compound **7**, BOP, DIPEA, CH_2_Cl_2_, rt, 2.5 h; f) CH_2_Cl_2_/TFA (3:1), rt, 3 h; g) MeOH/THF (1:1), NaOH (1 M), rt, 13 h.

The synthesis of a sorbitol containing CuAAC substrate for “Click-to-Chelate” started with the alkylation of 2,3;4,5-Di-O-isopropylidene D-glucitol (**1**) at the primary hydroxyl group with tert-butyl 4-bromobutanoate (**2**) by a reported 2-step procedure (Scheme 1) [22]. Removal of the acetonide groups of sorbitol derivative **3** followed by acetylation of the resulting pentaol **4** and subsequent cleavage of the tertBu-ester of intermediate **5** gave pentaacetate **6** in satisfying overall yield. Coupling of pentaacetate **6** to the N(ε)-amine of the previously reported propargyl lysine derivative **7** [17] provided product **8** in good yield. Stepwise deprotection of compound **8** yielded first intermediate **9**, which upon saponification with NaOH gave the desired glycated and deprotected alkyne synthon **10** for subsequent CuAAC.

**Scheme 2:**
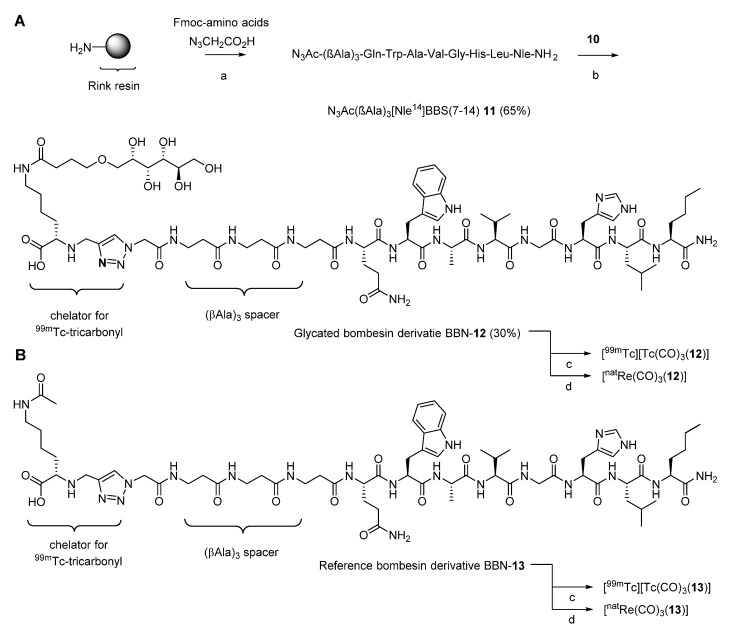
Peptide synthesis, CuAAC and (radio)metal complexation of sorbitol-containing peptide conjugate BBN-**12** (**A**) and reference compound BBN-**13** (**B**). a) SPPS: 1) Fmoc cleavage: 20% piperidine in DMF, rt; 2) Elongation: Fmoc-amino acid or azido acetic acid (N_3_CH_2_CO_2_H) respectively, HATU, DIPEA, DMF, 2 h, rt; 3) Cleavage and deprotection: TFA/H_2_O/PhOH/*i*Pr_3_SiH: 92.5:2.5:2.5:2.5, 2 h, rt; b) for BBN-**12**: compound **10**, Cu(OAc)_2_, Na-ascorbate, CH_3_CN/H_2_O, rt, 13 h; for BBN-**13**: see reference [23]; c) [^99m^Tc][Tc(CO)_3_(H_2_O)_3_]^+^, 100° C, 30 min; d) [N(Et)_4_]_2_[Re(CO)_3_Br_3_]_,_ 100 °C, 1 h.

Azido-bombesin derivative **11** was prepared manually by standard Fmoc solid phase peptide synthesis (SPPS) as previously described (Scheme 2A) [23]. In order to avoid potential interference of the radiometal complex with the tumor-targeting BBN vector, a spacer consisting of three βAla was introduced between the moieties. The peptide was cleaved from the resin, deprotected, and reacted in aqueous solution with alkyne derivative **10** using Cu(OAc)_2_ and Na-ascorbate. This yielded, after HPLC purification, the glycated peptide conjugate BBN-**12**. Reference compound BBN-**13**, identical in all aspects to the investigational compound BBN-**12** but lacking the carbohydrate moiety, was prepared by the same chemistry as previously reported [23].

### 2.2. (Radio) Metal-Labeling and Characterization

The tricarbonyl precursor [^99m^Tc][Tc(CO)_3_(H_2_O)_3_]^+^ was prepared by adding [^99m^Tc][TcO_4_]^−^ to the CRS kit (Center for Radiopharmaceutical Sciences, Paul Scherrer Institute, Villigen, Switzerland) and heating of the reaction mixture for 30 min at 100 °C. The radiolabeling of both peptide conjugates BBN-**12** and BBN-**13** with [^99m^Tc][Tc(CO)_3_(H_2_O)_3_]^+^ was carried out successfully either in a conventional heating block at 100 °C for 30 min or in a microwave reactor at 120 °C for 5 min (Scheme 2). The desired radiopeptides [^99m^Tc][Tc(CO)_3_(L)] (L = BBN-**12**, **13**) were obtained independent of the reaction conditions in a radiochemical yield and purity of ≥ 95% as determined by analytical γ-HPLC and γ-TLC. 

For characterization of the ^99m^Tc-labeled peptides, the non-radioactive analogous rhenium compounds [^nat^Re(CO)_3_(L)] (L = **12**, **13**) were prepared in H_2_O by reaction of conjugates BBN-**12** and BBN-**13** with [N(Et)_4_]_2_[Re(CO)_3_Br_3_] at 100 °C for 1 h (Scheme 2) [17,24]. The products were purified by HPLC and analyzed by mass spectrometry. The comparison between the UV-HPLC (λ = 240 nm) of the ^nat^Re-tricarbonyl peptides and the γ-HPLC of the corresponding ^99m^Tc-tricarbonyl compounds confirmed their identity (for HPLC chromatograms and mass spectrometric analysis see SI, Figures S12–S15). 

### 2.3. TLC Analytics

In order to have an additional quality control with easy and rapid performance, a TLC system was developed to analyze the radiolabeled peptides conjugates (Table 1). The following retention factors (R_f_) were obtained for each analyzed sample and the radiochemical purity of the radiolabeled peptides was always in accordance to the results obtained using the γ-HPLC method. The impurities identified were either unreacted pertechnetate (~0.7%) and/or ^99m^Tc-tricarbonyl precursor (~1.8%), which is also in accordance to the γ-HPLC results.

### 2.4. LogD

For logD analysis, the octanol/PBS partition coefficients of the ^99m^Tc-tricarbonyl labeled BBN conjugates were determined at pH 7.4 utilizing the “shake-flask method” [25,26]. The logD value for the reference compound [^99m^Tc][Tc(CO)_3_(BBN-**13**)] was −0.54 ± 0.04 while for the glycated compound [^99m^Tc][Tc(CO)_3_(**12**)] it was −1.26 ± 0.16 (Table 2). As expected, these measurements confirmed the increased hydrophilicity of the glycated ^99m^Tc-labeled BBN conjugate due to the introduction of the sorbitol moiety. The physicochemical properties of the ^99m^Tc-labeled peptides are summarized in Table 2.

### 2.5. In Vitro Evaluation

**Figure 2:**
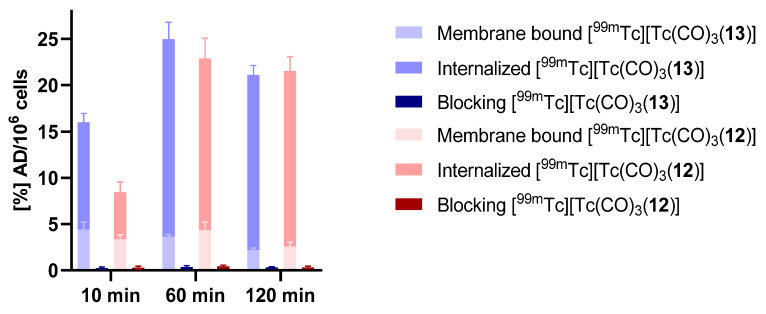
Cell binding and internalization of radiolabeled bombesin derivatives [^99m^Tc][Tc(CO)_3_(**12**)] and [^99m^Tc][Tc(CO)_3_(**13**)] using GRP-receptor expressing PC-3 cells; non-specific binding was determined with 1000-fold excess of BBN(1–14). Values are the mean ± SD (*n* = 2–3 in triplicate) and data are normalized to 10^6^ cells per well.

[^99m^Tc][Tc(CO)_3_(**12**)] was evaluated in vitro and compared with the reference compound [^99m^Tc][Tc(CO)_3_(**13**)]. Receptor binding and cell internalization properties of the ^99m^Tc(CO)_3_-labeled BBN derivatives were investigated with GRPR expressing PC-3 cells (*n* = 2–3 in triplicate). Despite the slightly slower uptake kinetics of [^99m^Tc][Tc(CO)_3_(**12**)], both radiolabeled compounds [^99m^Tc][Tc(CO)_3_(L)] (L = **12, 13**) showed a comparable extent of cell binding and internalization: A plateau was reached after 1 h (22.9% ± 0.4% and 25.0% ± 0.1% of the applied radioactivity (AD)/10^6^ cells, respectively; Table 2, Figure 2). The membrane bound fraction was less than 5% AD/10^6^ cells for both compounds. The receptor-specific cell uptake was verified by incubating the cells with the radiopeptides in the presence of 1000-fold excess of natural bombesin as blocking agent. For both radiolabeled peptides this resulted in a significant decrease of cellular binding and uptake (<0.5% AD/10^6^ cells).

**Figure 3:**
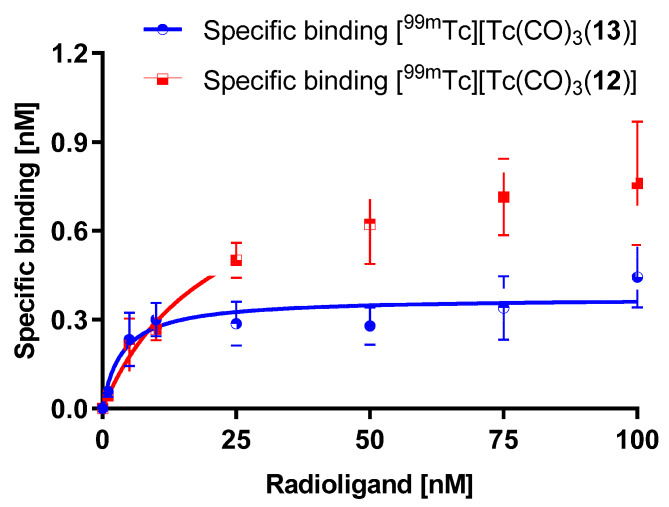
Results of receptor saturation experiments with radiolabeled bombesin (BBN) derivatives [^99m^Tc][Tc(CO)_3_(**12**)] and [^99m^Tc][Tc(CO)_3_(**13**)] using GRPR expressing PC-3 cells. K_d_ and B_max_ values were determined by nonlinear regression using GraphPad Prism 7. Data are the mean ± SD (*n* = 2–3 in triplicate) and normalized to 10^6^ cells per well.

The receptor affinity (K_d_) and maximum receptor occupancy (B_max_) were determined by receptor saturation assays using increasing amounts of the radiolabeled peptides (Figure 3). Receptor specificity of both compounds was demonstrated in the presence of a large excess of natural BBN. Higher values for K_d_ and B_max_ were observed for [^99m^Tc][Tc(CO)_3_(**12**)] (K_d_ = 21.3 ± 4.55 nM, B_max_ = 0.91 ± 0.06 nM) in comparison with the reference compound [^99m^Tc][Tc(CO)_3_(**13**)] (K_d_ = 3.7 ± 1.0 nM, B_max_ = 0.37 ± 0.02 nM; Table 2) [23]. Some variability in experimentally determined K_d_ and B_max_ of structurally related radiolabeled peptides is not uncommon [18,23,27], yet in this case somewhat surprising as we did not expect the sorbitol moiety to have an influence on these parameters; this in particular because the results of cell binding and internalization experiments were similar for both radiolabeled peptides (Figure 2). Differences in specific molar activities (MBq/mol) could account for this observation and cannot be entirely excluded even though both ^99m^Tc-radiopeptides were prepared under identical conditions. Because this study aims at the investigation of differences in the pharmacokinetics of the radiopeptides (e.g., rate and route of excretion) rather than in tumor uptake, we continued with the evaluation of both compounds in vivo. 

### 2.6. In Vivo Evaluation

**Figure 4:**
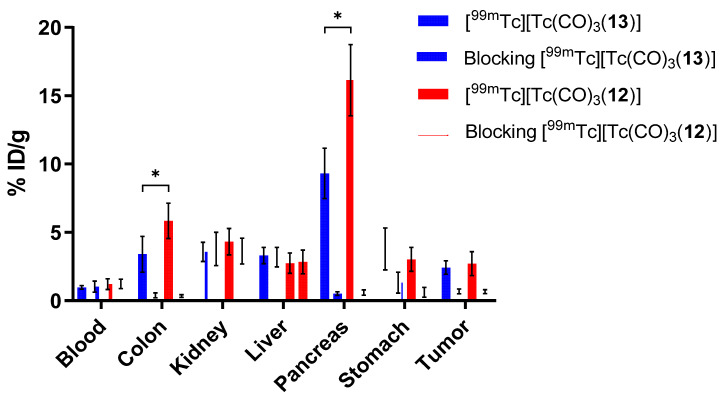
Biodistribution of radiolabeled bombesin derivatives [^99m^Tc][Tc(CO)_3_(**12**)] and [^99m^Tc][Tc(CO)_3_(**13**)] under blocking conditions in selected organs and tumors of mice bearing PC-3 cell xenografts at 1 h p.i. (*n* = 5–6); results are expressed as the percentage of injected dose per gram of tissue (%ID/g); error bars indicate standard error of the mean; * indicates α < 0.05.

Biodistribution studies were performed in nude mice bearing PC-3 tumor xenografts at 1 h post-injection (p.i.) of the radiotracers (*n* = 6; Table 3, Figure 4). 2000-fold excess of BBN(1–14) was used as blocking agent and co-injected with the radiolabeled conjugate [^99m^Tc][Tc(CO)_3_(**13**)] or [^99m^Tc][Tc(CO)_3_(**12**)].

The blood clearance of both radiopeptides [^99m^Tc][Tc(CO)_3_(L)] (L = **12**, **13**) was very fast with 0.98% ± 0.14% and 1.22% ± 0.40% ID/g, respectively, already at 1 h p.i. Glycated [^99m^Tc][Tc(CO)_3_(**12**)] showed a significantly higher specific uptake than the reference compound [^99m^Tc][Tc(CO)_3_(**13**)] in the GRPR positive pancreas and in the colon. Very low accumulation of radioactivity was observed in receptor-negative tissues and organs (e.g., the uptake in muscle and bone was less than 0.5% ID/g, Table 3). Blocking experiments resulted in a significant decrease of accumulation of radioactivity in GRPR-positive tumors and organs therefore demonstrating receptor specific uptake of the radiotracer (*n* = 5; Table 3, Figure 4). No significant differences in tumor uptake were observed as expected based on the in vitro cell binding and internalization experiments (Figure 2, Table 2). Despite glycation, the pharmacokinetics of [^99m^Tc][Tc(CO)_3_(**12**)] was not significantly altered in comparison to [^99m^Tc][Tc(CO)_3_(**13**)] and the enhanced renal excretion and lowered uptake in the liver was not as pronounced as expected. Therefore, the glycated radiopeptide did not perform significantly better in vivo. Nevertheless, as hypothesized, [^99m^Tc][Tc(CO)_3_(**12**)] bearing a sorbitol moiety did not show a pronounced accumulation in liver and spleen compared to the previously published glucuronated BBN derivative [18].

## 3. Materials and Methods 

Caution: ^99m^Tc is a γ-emitter (140 keV) with a half-life of 6.01 h. All reactions involving ^99m^Tc were performed in a laboratory approved for the handling of radionuclides and appropriate safety procedures were followed at all times to prevent contamination.

### 3.1. General Procedures

Solvents and all other chemicals of at least of synthesis grade were purchased from B. Braun, Sigma-Aldrich, and Bachem. Buffers and stock solutions were prepared using Millipore water. [^99m^Tc]NaTcO_4_ was eluted from an Ultra-TechneKow or TEKCIS ^99^Mo/^99m^Tc generator. The precursor [^99m^Tc][Tc(CO)_3_(H_2_O)_3_]^+^ was prepared by using the CRS kit for ^99m^Tc-tricarbonyl (Center for Radiopharmaceutical Sciences, Paul Scherrer Institute, Villigen, Switzerland). The radiolabeling of the peptides was performed with a Biotage^®^ Initiator+ microwave using a 200–500 µL glass reactor or with a heating block in 1.5 mL LoProtein bind Eppendorf vessels. HPLC analyses were carried out with an Agilent system (Vienna, Austria) equipped with Autosampler Agilent 1100 Series, Iso Pump (Isocratic Pump) Agilent 1200 Series G1310A, UV-Monitor Agilent 1200 Series G1314B Variable Wavelength Detector (VWD) and Radioactivity Detector Elysia Raytest Gabi Star. Data acquisition and gradient control were performed using GINA StarTM, version 5.9. HPLC solvents were 0.1% TFA in H_2_O (A) and MeCN (B). Quality control of the radiometal labeled peptide was performed using a C12 Phenomenex Jupiter column (4u Proteo 90 Å, 4 µm, 250 × 4,6 mm) and a linear gradient from 80% to 50% of eluent A in 16 min with a flow rate of 1 mL/min. Low resolution (LR)-MS was performed on a Bruker maXis (UHR-TOF, Vienna, Austria) equipped with ESI ion source and Qq-TOF analyser or Bruker amaZon speed ETD supplied with ESI ion source and 3D ion trap. Sample centrifugation was done by Hettich Universal 30 RF with Hettich rotor 1412 24 × 3*g*. 

In vitro and in vivo experiments were carried out with radiolabeled peptides with a purity of at least 95% (γ-HPLC). Samples were γ-counted for 30 sec using an energy window of 104–170 keV (2480 Wizard^2^, PerkinElmer, Waltham, Mass., USA).

### 3.2. Syntheses

Synthesis of compound **3**. The compound was prepared according to a published procedure ref. [22]. 2,3;4,5-Di-O-isopropylidene D-glucitol (1, 525 mg, 2 mmol) and Bu_2_SnO (550 mg, 2.2 mmol) were suspended in toluene (20 mL) and stirred at reflux with a Dean Stark for 24 h. Tert-butyl 4-bromobutanoate (2, 888 mg, 4 mmol) and tertrabutylammonium iodide (148 mg, 0.4 mmol) were added to the solution and stirring at reflux was continued for 24 h. The solvent was removed *in vacuo* and the crude product purified by chromatography on silicagel with ethyl acetate/hexane (1:4 → 1:2) to yield product **3** as a yellow oil (250 mg, 31%). ^1^H-NMR (CDCl_3_): δ = 4.10 (dd, 1H, *J* = 5.7 and 8.3 Hz), 4.05–3.96 (m, 2H), 3.95–3.91 (m, 2H), 3.90–3.88 (m, 1H), 3.55–3.45 (m, 4H), 2.45 (d, 1H, *J* = 7.1 Hz), 2.28 (t, 2H, *J* = 7.3 Hz), 1.88–1.80 (m, 2H), 1.41 (s, 9H), 1.38 (s, 3H), 1.382 (s, 3H), 1.34 (s, 3H), 1.30 (s, 3H) ppm; ^13^C-NMR (CDCl_3_): δ = 172.9, 109.8, 109.6, 80.3, 80.2, 77.3, 77.1, 72.8, 70.5, 69.1, 67.9, 32.4, 28.2, 27.3, 26.9, 26.7, 25.4, 25.2 ppm; HR-MS: [M + Na^+^]^+^ = 427.2299, calcd for C_20_H_36_O_8_Na: 427.2308.

Synthesis of pentaol **4**. Attempted deprotection of bis-acetonide **3** (404 mg, 1 mmol) in aq. AcOH (80%, 5 mL) by stirring at rt overnight did not provide expected pentaol **4** but quantitative formation of a single new product which corresponded to a not further characterized mono-acetonide (360 mg, quant.; LR-MS, [M + Na^+^]^+^ = 387.4, calcd for C_17_H_32_O_8_Na: 387.2). Upon standing at rt in CHCl_3_ (or CH_2_Cl_2_) the mono-acetonide intermediate disproportionated to a mixture of isomers consisting of bis- and mono-acetonides (LR-MS) and fully deprotected pentaol **4**. The latter was isolated by evaporation of the solvent under reduced pressure and purification by flash chromatography on silicagel with CH_2_Cl_2_/MeOH (9:1→5:1). The mixture of bis-and mono-acetonides was also isolated and subjected to an additional cycle of CHCl_3_^–^ mediated isomerization to yield pentaol **4** as a pale yellow oil (100 mg, total yield 33% (2 cycles) from compound **3**). LR-MS: [M + Na^+^]^+^ = 347.16, calcd for C_14_H_28_O_8_Na: 347.17. The compound was fully characterized by NMR as its pentaacetate **5** (see below).

Synthesis of pentaacetate **5**. Pentaol **4** (77 mg, 0.24 mmol) was dissolved in CH_2_Cl_2_ (1 mL) and acetic acid anhydride (0.5 mL) and pyridine (0.3 mL) were added. The solution was stirred at rt for 18 h. Evaporation in vacuo and purification by flash chromatography on silicagel with CH_2_Cl_2_/MeOH (9:1→5:1) gave pentaacetate tBu-ester **5** as a colorless oil (110 mg, 96%).^1^H-NMR (CDCl_3_): δ = 5.53 (dd, 1H, *J* = 7.1 and 3.5 Hz), 5.40 (dd, 1H, *J* = 7.6 and 3.5 Hz), 5.12–5.02 (m, 2H), 4.23 (dd, 1H, *J* = 12.4 and 3.3 Hz), 4.11 (dd, 1H, *J* = 12.4 and 5.3 Hz), 3.58 (dd, 1H, *J* = 11.0 and 4.6 Hz), 3.53–3.45 (m, 2H), 3.43–3.35 (m, 2H), 2.38–2.23 (m, 1H), 2.12 (s, 3H), 2.07 (s, 3H), 2.06 (s, 3H), 2.05 (s, 6H), 1.85–1.80 (m, 2H), 1.43 (s, 9H) ppm; ^13^C-NMR (CDCl_3_): δ = 172.8, 170.6, 170.3, 170.0, 169.9, 80.1, 71.0, 70.7, 68.9, 68.8, 68.7, 68.5, 61.7, 32.2, 28.2, 25.1, 21.0, 20.91, 20.86, 20.8, 20.7 ppm (1 carbonyl signal not observed due to overlapping signals); LR-MS: [M + Na^+^]^+^ = 557.29, calcd for C_24_H_38_O_13_Na: 557.54.

Synthesis of carboxylic acid **6**. Pentaacetate tertBu-ester **5** (110 mg, 0.21 mmol) was dissolved in CH_2_Cl_2_/TFA (3:1, 4 mL) and stirred at rt for 18 h. Evaporation of the volatile components under reduced pressure provided carboxylic acid **6** as a yellow oil (98 mg, quant.). ^1^H-NMR (CDCl_3_): δ = 5.54 (dd, 1H, *J* = 7.1 and 3.6Hz), 5.40 (dd, 1H *J* = 7.4 and 3.6Hz), 5.10–5.07 (m, 2H), 4.25 (dd, 1H *J* = 12.4 and 3.2 Hz), 4.12 (dd, 1H, *J* = 12.4 and 5.6 Hz), 3.60 (dd, 1H, *J* = 10.9 and 4.6 Hz), 3.55–3.43 (m, 3H), 2.50–2.43 (m, 2H), 2.13 (s, 3H), 2.074 (s, 3H), 2.069 (s, 3H),2.066 (s, 3H),2.061 (s, 3H), 1.96–1.86 (m, 2H) ppm; LR-MS: [M + Na^+^]^+^ = 501.16, calcd for C_20_H_30_O_13_Na: 501.16.

Synthesis of compound **8**. Pentaacetate **6** (96 mg, 0.2 mmol) was dissolved in CH_2_Cl_2_ (3 mL) and Lys-derivative 7 [17] (77 mg, 0.26 mmol), BOP (134 mg, 0.3 mmol), and DIPEA (0.3 mL, 1 mmol) were added. The solution was stirred at rt for 2.5 h. Evaporation under reduced pressure and purification of the crude product by flash chromatography on silicagel with EtOAc gave coupling product **8** as a pale yellow oil (140 mg, 92%). LR-MS: [M + H^+^]^+^ = 759.36, calcd for C_35_H_55_N_2_O_16_: 759.38. As already reported [17], the formation of rotamers due to the presence of the trisubstituted N(α)amine of the Lysine resulted in the duplication and/or broadening of NMR signals; thus, the compound was fully characterized after the Boc-deprotection (see compound **9**). 

Synthesis of pentaacetate **9**. Compound **8** (80 mg, 0.11 mmol) was dissolved in CH_2_Cl_2_/TFA (3:1, 4 mL) and kept at rt for 3 h after which time TLC indicated completed conversion. The solution was evaporated under reduced pressure, the residue was dissolved in MeOH (2 mL), filtered through Celite^TM^ and dried in vacuo to provide the TFA salt of compound **9** as a colorless oil (75 mg, 93%). ^1^H-NMR (MeOH-d_4_; recorded after complete H/D exchange): δ = 5.51 (dd, 1H, *J* = 4.0 and 6.3Hz), 5.41 (dd, 1H, *J* = 4.0 and 6.9 Hz), 5.16–5.10 (m, 2H), 5.08–5.03 (m, 2H), 4.31 (dd, 1H, *J* = 12.3 and 3.4 Hz), 4.18 (dd, 1H, *J* = 7.0 and 5.0 Hz), 4.12 (dd, 1H, *J* = 12.3 and 5.7 Hz), 4.02 (t, 2H, *J* = 2.5 Hz), 3.87 (s, 3H), 3.58 (dd, 1H, *J* = 11.0 and 5.0 Hz), 3.54–3.40 (m, 3H), 3.28 (t, 1H, *J* = 2.5 Hz), 3.19 (t, 2H, *J* = 6.9 Hz), 2.27 (t, 2H, *J* = 7.3 Hz), 2.17 (s, 3H), 2.05 (s, 3H), 2.04 (s, 3H), 2.03 (s, 3H), 2.02 (s, 3H), 2.11–1.95 (m, 2H), 1.90–1.80 (m, 2H), 1.62–1.50 (m, 2H), 1.50–1.31 (m, 2H) ppm; ^13^C-NMR (MeOH-d4): δ = 175.8, 172.2, 171.8, 171.59, 171.56, 171.5, 160.4 (q, JC-F = 39 Hz), 116.9 (q, JC-F = 277 Hz), 79.8, 74.1, 72.0, 71.7, 70.4, 70.2, 70.1, 69.8, 62.7, 59.9, 54.0, 39.6, 36.4, 33.7, 29.9, 29.8, 27.0, 22.9, 20.81, 20.80, 20.63, 20.62, 20.6 ppm; LR-MS: [M + H^+^]^+^ = 659.33, calcd for C_30_H_47_N_2_O_14_: 659.33.

Synthesis of compound **10**. Pentaacetate **9** (77 mg, 0.1 mmol) was dissolved in MeOH/THF (1:1, 1 mL) and NaOH (1 M, 2 mL) was added. The solution was stirred at rt for 13 h and the pH was adjusted to pH 4 by addition of HCl (1 M). The solution was concentrated under reduced pressure and the residue was triturated in dry isopropanol (2 mL). The salts were filtered off and the filtrate was evaporated in vacuo to provide compound **10** as a colorless oil (39 mg, 91%). ^1^H-NMR (MeOH-d_4_, recorded after complete H/D exchange): δ = 3.89 (dd, 1H, *J* = 16.6 and 2.6 Hz), 3.81 (dd, 1H, *J* = 16.6 and 2.6 Hz), 3.81–3.77 (m, 2H), 3.70 (dd, 1H, *J* = 11.0 and 3.3 Hz), 3.67–3.61 (m, 2H), 3.598–3.53 (m, 2H), 3.52–3.34 (m, 6H), 3.10 (t, 2H, *J* = 6.5 Hz), 3.08 (t, 1H, *J* = 2.5 Hz), 2.20 (t, 2H, *J* = 7.3 Hz), 1.92–1.70 (m, 4H), 1.52–1.29 (m, 4H) ppm; ^13^C-NMR (MeOH-d_4_): δ = 176.0, 172.7, 79.3, 79.2, 74.7, 73.2, 73.0, 72.7, 71.3, 70.9, 64.5, 61.9, 39.8, 36.7, 33.8, 30.6, 29.9, 26.9, 23.2 ppm; LR-MS, [M + H^+^]^+^ = 435.4, calcd for C_19_H_35_N_2_O_9_: 435.23; LR-MS: [M + Li^+^]^+^ = 441.4, calcd for C_34_H_47_N_2_O_9_Li: 441.24.

Synthesis of azido peptide **11**. The synthesis of azido peptide **11** has been previously described [23]. For manual solid phase peptide synthesis, Fmoc-protected amino acids (Fmoc-Gln(Trt)-OH, Fmoc-Trp(Boc)-OH, Fmoc-Ala-OH, Fmoc-Val-OH, Fmoc-Gly-OH, Fmoc-His(Trt)-OH, Fmoc-Leu-OH, Fmoc-Nle-OH) or azidoacetic acid (3 equiv.) were coupled to a Rink resin (scale: 0.025 mmol) in a syringe fitted with a polypropylene frit and a teflon tap in the presence of HATU (3 equiv.) and DIPEA (5 equiv.) in DMF for 2 h. The completion of the reaction was verified by the Kaiser test and repeated if necessary. Elongation yields were determined by the UV-absorption measurement of the fluorenylmethylpiperidine adduct after treatment of the resin with 5 mL of a 20% piperidine/DMF solution (3 × 3 min). Azido peptide **11** was cleaved from the resin and deprotected using a solution of trifluoroacetic acid, phenol, water and triisopropylsilane (1 mL; 92.5/2.5/2.5/2.5%) as a cleavage cocktail at rt for 2 h. After precipitation in ice-cold diethyl ether, the crude peptide was recovered by centrifugation and washed twice with cold diethyl ether. The precipitate was dissolved in water (5–10 mg/mL), lyophilized and analyzed by HPLC and MS: white fluffy powder (20 mg, 65%), purity according to HPLC: >80%, LR-MS: [M + H^+^]^+^ = 1219.32, calcd for C_55_H_84_N_19_O_13_: 1219.40.

Synthesis of glycated peptide BBN-**12**. Crude azido peptide **11** (13 mg; purity: ca. 80%; 8 µmol) was dissolved in 20% CH_3_CN/H_2_O (1 mL) and Cu(OAc)_2_ (1 equiv.), sodium ascorbate (2 equiv.), and alkyne-sorbitol building block 10 (5.5 mg, 1.5 equiv.) were added. The reaction mixture was stirred at rt for 13 h, filtered, and purified by semi-preparative HPLC (gradient: 23–35% 0.1% TFA in CH_3_CN within 15 min; flow 4 mL/min, λ = 215 nm). After liophylization, peptide BBN-**12** was isolated as a white fluffy powder (4 mg, 30%), purity according to HPLC: >95%, LR-MS: [M + H^+^]^+^ = 1652.99, calcd for C_74_H_118_N_21_O_22_: 1652.876.

### 3.3. Radiolabeling

#### 3.3.1. [^99m^Tc][Tc(CO)_3_(H_2_O)_3_]^+^

Technetium was provided in form of [^99m^Tc][TcO_4_]^−^ from a ^99^Mo/^99m^Tc generator. The generator was eluted at least 24 h before the elution for the labeling in order to obtain sufficient specific activity of the pertechnetate. The eluate contained 1–2 GBq/mL in 0.9% saline. [^99m^Tc][Tc(CO)_3_(H_2_O)_3_]^+^ was prepared by the procedures described in the instruction leaflet of the CRS kit. In brief, 1.0–1.2 mL of [^99m^Tc][TcO_4_]^−^ solution was added to the CRS kit for tricarbonyl and subsequently heated for 30 min at 100 °C. Afterwards, the solution (pH > 10) was cooled to room temperature and neutralized to pH 7 with an acidified phosphate buffer (1:2 mix of 1 M Na_2_HPO_4_/NaH_2_PO_4_ (1:1):1 M HCl; pH 1.5, 50 µL). The radiochemical yield and purity of [^99m^Tc][Tc(CO)_3_(H_2_O)_3_]^+^ was determined by γ-HPLC (R_t_ = 10.35 min) and γ-TLC (for R_f_ values see SM, Figures S12–S14) and was ˃98%.

#### 3.3.2. [^99m^Tc][Tc(CO)_3_(L)] (L = **12**, **13**)

20 µL of the respective stock solution of the peptide conjugates in water (compound BBN-**12** or BBN-**13**; 1 mM, 20 nmol) were added to 180 µL of ^99m^Tc-tricarbonyl solution (~150 MBq, 0.1 mM final peptide concentration). The reaction was completed either by heating at 100 °C for 30 min in a heating block or at 120 °C for 5 min in a microwave reactor. The identity and radiochemical purity of [^99m^Tc][Tc(CO)_3_(L)] (L = **12**, **13**) was determined by γ-HPLC (R_t ([99mTc][Tc(CO)3(13)])_ = 13.05 min, R_t ([99mTc][Tc(CO)3(12)])_ = 11.52 min; see SM, Figures S12 and S13) and γ-TLC (for R_f_ values see Table 1 and SM, Figure S16) and was at least 95%. 

#### 3.3.3. [^nat^Re(CO)_3_(L)] (L = **12**, **13**)

[^nat^Re(CO)_3_(L)] (L = **12**, **13**) were prepared by mixing 20 µL of aqueous stock solutions containing peptide BBN-**12** or BBN-**13** (1 equiv, 1 mM) with 30 µL of [N(Et)_4_]_2_[Re(CO)_3_Br_3_] (1.5 equiv, 1 mM) in water [23]. The reaction was heated at 100 °C for 1 h. The products were purified by HPLC and analyzed by LR-MS. [^nat^Re(CO)_3_(13)]: R_t_ = 12.58 min (see SM, Figure S12); LR-MS: [M + H^+^]^+^ = 1714.72 (calcd for C_69_H_100_N_21_O_19_ Re: 1714.71; see SM, S14). [^nat^Re(CO)_3_(12)]: R_t_ = 11.47 min (see SM, Figure S13); LR-MS: [M + H^+^]^+^ = 1922.80 (calcd for C_77_H_116_N_21_O_25_ Re: 1922.80; see SM, Figure S15).

### 3.4. TLC 

The purity of the ^99m^Tc-labeled peptides was additionally confirmed by thin-layer chromatography (TLC) using silica gel 60 F_254_ aluminum plates (Merck, Darmstadt, Germany), as stationary phase (strips height 10 cm with 8 cm between application point and solvent front). A mixture 95.5:0.5 of MeOH:HCl 6 M was used as mobile phase. In a typical assay, 2 µL drops of freshly prepared [^99m^Tc][Tc(CO)_3_(**13**)] or its glycated derivative [^99m^Tc][Tc(CO)_3_(**12**)] were spotted on the plates. After drying the spots, the plate was developed and subsequently dried. Likewise, samples of [^99m^Tc]TcO_4_^−^ and [^99m^Tc][Tc(CO)_3_(H_2_O)_3_]^+^ were analyzed in order to identify the ^99m^Tc-radiolabeled peptides. The readout of the radio-TLC was performed using a Canberra-Packard Instant Imager (Packard Instrument Company, Meriden, USA) using Imager software (version 2.05 for Windows 95). Regions of interests (ROIs) were defined for all radioactivity peaks found along the plate while start and front parameters of the TLC-plate were given in the software. Consequently, R_f_ values were determined and the radiochemical purity of the radiotracers calculated as the percentage of the counts of [^99m^Tc][Tc(CO)_3_(**13**)] (*n* = 3 in duplicates) or [^99m^Tc][Tc(CO)_3_(**12**)] (*n* = 2 in duplicates) divided by the sum of counts in all ROIs.

### 3.5. LogD

The lipophilicities (LogD) of [^99m^Tc][Tc(CO)_3_(L)] (L = **12**, **13**) were determined by their partition coefficient between n-octanol and PBS (pH 7.4) utilizing the “shake-flask method” [25,26]. PBS and n-octanol were shaken overnight to saturate each phase. After separation of the layers by gravity, equal volumes (500 µL) of each layer were taken and transferred into an Eppendorf tube and 5 µL (~45 kBq) of the radiolabeled peptide solution were added to the PBS/n-octanol mixture. The resulting solutions were mixed in a shaker at room temperature for 20 min and centrifuged at 3000 rpm for 10 min. Aliquots of 300 µL were removed from the octanol and PBS phases and the radioactivity was measured in the γ-counter (*n* = 3 in triplicates). The lipophilicity was calculated as the log value of the average ratio between the radioactivity in the organic fraction (octanol) and the PBS fraction from the samples.

### 3.6. In Vitro Evaluation

#### 3.6.1. General Methods

Cell culture reagents were purchased from Thermo Fisher Scientific (Vienna, Austria) or Sigma-Aldrich (Vienna, Austria). Human prostate adenocarcinoma cells (PC-3) were purchased from ATCC and cultivated in RPMI-1640 medium supplemented with 10% heat-inactivated fetal bovine serum (FBS), 2 mM L-glutamine, 100 units/mL penicillin and 100 μg/mL streptomycin under humidified atmosphere (37 °C, 5% CO_2_) until minimum 80% at confluence. In order to determine the number of cells for each experiment, three extra wells were seeded for parallel cell counting using the Neubauer chamber (Ratiomed) and Trypan Blue solution, 0.4% (Gibco^®®^). Percentage of applied dose was normalized to 10^6^ cells/well.

#### 3.6.2. Internalization Studies

Internalization studies were performed as previously published [23]. In brief, approximately 10^6^ PC-3 cells were seeded in 1% FBS RPMI-medium (RPMI-1640 medium containing 1% FBS, 2 mM L-glutamine, 100 units/mL penicillin and 100 μg/mL streptomycin) in 6-well plates on the day before the experiment and incubated overnight in humidified incubator (37 °C, 5% CO_2_). Approx. one hour before the experiment, the medium was replaced with 1.3 mL fresh 1% FBS RPMI-medium. Subsequently, 100 µL of radiolabeled peptide [^99m^Tc][Tc(CO)_3_(L)] (L = **12**, **13**; 0.25 pmol; ~1.0 kBq) were added to each well and cells were incubated for different time points (10, 60, and 120 min). Non-specific receptor binding was determined by incubating the cells with the radiotracers and 1000-fold excess of natural bombesin (250 pmol; 100 μL per well) as the receptor blocking agent. After the respective incubation time, the supernatant was collected and the cells were washed twice with 1 mL ice-cold DPBS (Dulbecco’s phosphate-buffered saline). The combined fractions represent the unbound radiopeptide. The receptor-bound radioactivity was obtained by incubating the cells twice for 5 min with an ice-cold acidic glycine solution (100 mM NaCl, 50 mM glycine, pH 2.8; 1 mL) on ice followed by removal of the supernatant. Finally, the internalized fraction was collected after cell lysis using 1 M NaOH (1 mL; 10 min; 37 °C, 5% CO_2_) and the wells containing the cell lysate were washed twice with NaOH (1 M, 1 mL). Standards of the radiolabeled peptides [^99m^Tc][Tc(CO)_3_(L)] (L = **12**, **13**; per well: 100 μL; 0.25 pmol; ~1.0 kBq) for determination of the applied dose were prepared in triplicate. All fractions were measured in a γ-counter and calculated as percentage of the applied dose (*n* = 2–3 in triplicate).

#### 3.6.3. Receptor Saturation Assay

Receptor binding assays were performed as previously published [23]. PC-3 cells were prepared in 6-well plates as described above for the internalization experiments. Approx. one hour before the experiment, the medium was replaced with 0.8 mL fresh 1% FBS RPMI-medium. PC-3 cells were kept on ice for 30 min to stop receptor internalization processes prior to the start of the experiment. Afterwards, the cells were incubated at 4 ˚C with increasing concentration (1, 5, 10, 25, 50, 75, and 100 nM in NaCl; 100 μL/well, 5–600 kBq) of the radiolabeled peptides [^99m^Tc][Tc(CO)_3_(L)] (L = **12**, **13**) to allow receptor saturation. Non-specific receptor binding was determined using an excess of natural bombesin (2.5 μM/well for concentrations of the radiopeptide <10 nM, and 10 μM/well for higher concentrations). After incubation at 4 °C for 2 h, the supernatant was collected and the cells were washed twice with 1 mL of ice-cold DPBS. The combined fractions represent the unbound radiopeptide. To determine the cell-bound fraction, the cells were lysed with 1 M NaOH (1 mL; 10 min; 37 °C, 5% CO_2_) and the wells were washed twice with 1 M NaOH (1 mL). The obtained fractions were measured in a γ-counter. Dissociation constants (K_d_) and maximum receptor occupancy (B_max_) were calculated from the data for specific binding with nonlinear regression using GraphPad Prism 7 (*n* = 2–3 in triplicate).

### 3.7. In Vivo Evaluation

#### Biodistribution Experiments

All animals were treated according to the European Union rules on animal care. Animal experiments were approved by the Austrian Ministry of Sciences (BMBWF-66.009/0122-V/3b/2019). Approximately 5 × 10^6^ PC-3 cells in 100 µL serum-free medium were subcutaneously injected into flanks of 8-week-old, female, athymic mice (Fox-1^nu^, Charles River Laboratories). Xenografts were allowed to grow for 16 days (approx. size: 250 mm^3^) and mice were intravenously injected via the tail vein with 0.1 µM radiolabeled peptide (10 pmol, ~42 kBq, 100 µL physiological saline) alone (baseline experiments) or co-injected with 2000-fold excess BBN(1–14) (20 nmol; blocking experiments). One hour p.i. of the radiotracers, mice were sacrificed and organs, tumor and blood were removed. Radioactivity in organs and tissues was quantified using a γ-counter (2480 Wizard^2^, PerkinElmer). Organs and tissues were wet-weighted and percentage of injected dose per gram was calculated (% ID/g). Statistical analysis (2-way ANOVA) was performed by GraphPad Prism.

## 4. Conclusions

We have applied the “Click-to-Chelate” methodology to the efficient synthesis of a ^99m^Tc-tricarbonyl labeled tumor-targeting BBN conjugate that contains sorbitol as an acyclic, reduced carbohydrate modifier for increasing the hydrophilicity of the radiopeptide and, as a result, to promote renal elimination in vivo. Indeed, addition of sorbitol to the ^99m^Tc-labeled BBN derivative increased its hydrophilicity (logD) by 0.7 log units in comparison to a non-glycated reference compound. However, both compounds showed comparable pharmacokinetic profiles in vivo with regards to rate and route of excretion. For example, unspecific uptake of the radiotracer in the excretory organs kidneys and liver was found almost identical for both compounds despite the increased hydrophilicity of the glycated peptide. This puzzling result might be explained by the inherent properties of the studied peptide, [Nle^14^]BBN(7–14), and/or its metabolites. Indeed, low unspecific uptake in the liver has been reported for other related agonistic BBN derivatives radiolabeled with the ^99m^Tc-tricarbonyl core [28]. However, this phenomenon will need to be verified with other peptides or (bio)molecules of interest for nuclear medicine. Further investigations in this direction are currently ongoing and will be reported in due time.

The herein reported sorbitol-containing lysine derivative 10 (Scheme 1) represents a promising general alkyne precursor for the “Click-to-Chelate” approach which might proof useful for overcoming the often stated but yet unresolved issue of the increased lipophilicity of radiotracers resulting from the radiolabeling with the ^99m^Tc-tricarbonyl core [6].

## Figures and Tables

**Table 1:** TLC results for the quality control of the radiolabeled conjugates [^99m^Tc][Tc(CO)_3_(L)] (L = **12**, **13**; *n* = 2–3 in duplicates). Silica gel 60 F_254_ aluminum plates were used as stationary phase and a mixture 95.5:0.5 of MeOH:HCl 6 M as mobile phase. HPLC results are also reported for comparison.

Compound[a]	TLC		HPLC
	R_f_	Radiochemical Purity (%)	Radiochemical Purity (%)
[^99m^Tc]TcO_4_^-^	1.0	100	100
[^99m^Tc][Tc(CO)_3_(H_2_O)_3_]^+^	0.0	>95	>95
[^99m^Tc][Tc(CO)_3_(**13**)]	0.30–0.35	94.4 ± 0.2	95 ± 0.1
[^99m^Tc][Tc(CO)_3_(**12**)]	0.30–0.35	93.1 ± 0.5	94 ± 0.2

[a] Results from radiolabeling test experiments to demonstrate the conformity of the two methods for quality control; the products (<95% purity) were not used for biological experiments.

**Table 2:** Results from in vitro experiments with ^99m^Tc-radiopeptides.

Compound[a]	Total Cell Binding and Internalization % AD/10^6^ Cells[b]	K_d_ [nM][c]	B_max_ [nM][c]	logD[d]
[^99m^Tc][Tc(CO)_3_(**13**)]	25.0 ± 0.1	3.7 ± 1.0	0.37 ± 0.02	−0.54 ± 0.04
[^99m^Tc][Tc(CO)_3_(**12**)]	22.9 ± 0.4	21.3 ± 4.6	0.91 ± 0.06	−1.26 ± 0.16

[a] Compound [^99m^Tc][Tc(CO)_3_(**13**)] was reported previously [23] and results were comparable. [b] Total cell bound and internalized fraction of [^99m^Tc][Tc(CO)_3_(L)] (L = **12**, **13**) after 1 h. Results are normalized to 10^6^ PC-3 cells; values are the mean ± SD (*n* = 2–3 in triplicate). [c] Determined by receptor saturation binding assay; values are the mean ± SEM as calculated by GraphPad Prism 7 (*n* = 2–3 in triplicate). [d] Determined by the shake flask method; values are the mean ± SD (*n* = 3, in triplicate).

**Table 3:** Biodistribution data of radiolabeled peptide conjugates in nude mice bearing PC-3 xenografts.

Organ	[^99m^Tc][Tc(CO)_3_(13)] (% ID/g)[b]		[^99m^Tc][Tc(CO)_3_(12)] (% ID/g)[b]	
	1 h	1 h Blocked	1 h	1 h Blocked
Adrenal[a]	1.85 ± 0.67	0.46 ± 0.32	5.34 ± 1.85	0.54 ± 0.20
Blood	0.98 ± 0.14	1.03 ± 0.41	1.22 ± 0.40	1.24 ± 0.34
Bone	0.31 ± 0.07	0.52 ± 0.15	0.44 ± 0.28	0.63 ± 0.50
Brain	0.05 ± 0.01	0.06 ± 0.02	0.09 ± 0.07	0.07 ± 0.04
Colon[a]	3.40 ± 1.31	0.39 ± 0.18	5.85 ± 1.29	0.34 ± 0.11
Heart	0.32 ± 0.02	0.36 ± 0.14	0.50 ± 0.23	0.57 ± 0.12
Kidney	3.58 ± 0.70	3.79 ± 1.22	4.32 ± 0.97	3.64 ± 0.95
Intestine[a]	3.66 ± 1.60	1.11 ± 0.07	3.71 ± 1.48	0.69 ± 0.32
Liver	3.31 ± 0.60	3.19 ± 0.72	2.75 ± 0.74	2.85 ± 0.86
Lung	0.97 ± 0.30	1.03 ± 0.38	1.38 ± 0.83	1.69 ± 0.69
Muscle	0.30 ± 0.28	1.94 ± 1.08	0.35 ± 0.24	0.29 ± 0.04
Pancreas[a]	9.31 ± 1.84	0.52 ± 0.13	17.06 ± 3.25	0.60 ± 0.20
Spleen	1.16 ± 0.18	0.64 ± 0.13	1.16 ± 0.17	0.79 ± 0.32
Stomach[a]	3.79 ± 1.53	1.32 ± 0.77	3.03 ± 0.88	0.62 ± 0.35
Tumor[a]	2.43 ± 0.49	0.68 ± 0.17	2.72 ± 0.88	0.67 ± 0.14

[a] Gastrin-releasing peptide receptor (GRPR)-positive organs and tissues. [b] Results are given as the mean percentage of injected dose per gram of tissue (% ID/g) ± SD; n = 5–6 per group.

## Supplementary Materials

The following are available online at https://www.mdpi.com/1420-3049/25/11/2680/s1, ^1^H/^13^C-NMR and MS data of small molecules, UV-HPLC chromatograms and MS data of peptide conjugates, and γ-HPLC chromatograms as well as γ-TLC of ^99m^Tc-labeled peptide conjugates are available on-line.

## References

[1] Rösch F., Baum R.P. (2011). Generator-based PET radiopharmaceuticals for molecular imaging of tumours: On the way to THERANOSTICS. Dalt. Trans..

[2] Brandt M., Cardinale J., Aulsebrook M.L., Gasser G., Mindt T.L. (2018). An Overview of PET Radiochemistry, Part 2: Radiometals. J. Nucl. Med..

[3] García-Garayoa E., Schibli R., Schubiger A.P. (2007). Peptides radiolabeled with Re-186/188 and Tc-99m as potential diagnostic and therapeutic agents. Nucl. Sci. Tech..

[4] Boschi A., Uccelli L., Martini P. (2019). A picture of modern Tc-99m radiopharmaceuticals: Production, chemistry, and applications in molecular imaging. Appl. Sci..

[5] Alberto R., Ortner K., Wheatley N., Schibli R., Schubiger A.P. (2001). Synthesis and properties of boranocarbonate: A convenient in situ CO source for the aqueous preparation of [^99m^Tc(OH_2_)_3_(CO)_3_]^+^. J. Am. Chem. Soc..

[6] Kluba C.A., Mindt T.L. (2013). Click-to-Chelate: Development of technetium and rhenium-tricarbonyl labeled radiopharmaceuticals. Molecules.

[7] Kasten B.B., Ma X., Liu H., Hayes T.R., Barnes C.L., Qi S., Cheng K., Bottorff S.C., Slocumb W.S., Wang J. (2014). Clickable, hydrophilic ligand for fac-[MI(CO)_3_] + (M = Re/^99m^Tc) applied in an S-functionalized α-MSH peptide. Bioconjug. Chem..

[8] García-Garayoa E., Schweinsberg C., Maes V., Brans L., Bläuenstein P., Tourwé D.A., Schibli R., Schubiger A.P. (2008). Influence of the molecular charge on the biodistribution of bombesin analogues labeled with the [^99m^Tc(CO)_3_]-core. Bioconjug. Chem..

[9] Mindt T.L., Schweinsberg C., Brans L., Hagenbach A., Abram U., Tourwé D., García-Garayoa E., Schibli R. A click approach to structurally diverse conjugates containing a central di-1,2,3-triazole metal chelate. Chem. Med. Chem..

[10] Däpp S., García-Garayoa E., Maes V., Brans L., Tourwé D.A., Müller C., Schibli R. (2011). PEGylation of ^99m^Tc-labeled bombesin analogues improves their pharmacokinetic properties. Nucl. Med. Biol..

[11] Valverde I.E., Vomstein S., Fischer C.A., Mascarin A., Mindt T.L. Probing the backbone function of tumor targeting peptides by an amide-to-triazole substitution strategy. J. Med. Chem..

[12] Moradi S.V., Hussein W.M., Varamini P., Simerska P., Toth I. (2016). Glycosylation, an effective synthetic strategy to improve the bioavailability of therapeutic peptides. Chem. Sci..

[13] Schottelius M., Laufer B., Kessler H., Wester H.J. (2009). Ligands for mapping αvβ3-integrin expression in vivo. Acc. Chem. Res..

[14] Mindt T.L., Struthers H., Brans L., Anguelov T., Schweinsberg C., Maes V., Tourwe D., Schibli R. (2006). “Click to chelate”: Synthesis and installation of metal chelates into biomolecules in a single step. J. Am. Chem. Soc..

[15] Rostovtsev V.V., Green L.G., Fokin V.V., Sharpless K.B. (2002). A stepwise huisgen cycloaddition process: Copper(I)-catalyzed regioselective “ligation” of azides and terminal alkynes. Angew. Chem. Int. Ed..

[16] Tornøe C.W., Christensen C., Meldal M. (2002). Peptidotriazoles on solid phase:  [1,2,3]-triazoles by regiospecific copper(I)-catalyzed 1,3-dipolar cycloadditions of terminal alkynes to azides. J. Org. Chem..

[17] Mindt T.L., Struthers H., Spingler B., Brans L., Tourwé D., García-Garayoa E., Schibli R. (2010). Molecular assembly of multifunctional ^99m^Tc radiopharmaceuticals using “clickable” amino acid derivatives. Chem. Med. Chem..

[18] Römhild K., Fischer C.A., Mindt T.L. (2017). Glycated 99mTc-tricarbonyl-labeled peptide conjugates for tumor targeting by “click-to-chelate”. Chem. Med. Chem..

[19] Rangger C., Haubner R. (2020). Radiolabelled peptides for positron emission tomography and endoradiotherapy in oncology. Pharmaceuticals.

[20] Weinstein E.A., Ordonez A.A., DeMarco V.P., Murawski A.M., Pokkali S., MacDonald E.M., Klunk M., Mease R.C., Pomper M.G., Jain S.K. (2014). Imaging enterobacteriaceae infection in vivo with ^18^F-fluorodeoxysorbitol positron emission tomography. Sci. Transl. Med..

[21] Leamon C.P., Reddy J.A., Klein P.J., Vlahov I.R., Dorton R., Bloomfield A., Nelson M., Westrick E., Parker N., Bruna K. (2011). Reducing undesirable hepatic clearance of a tumor-targeted vinca alkaloid via novel saccharopeptidic modifications. J. Pharm. Exp..

[22] Jagadeesh Y., Reddy J.S., Rao V.B., Swarnalatha L.J. (2010). Total synthesis of (−)-codonopsinol and (+)-2-epi codonopsinol via acid catalyzed amido cyclisation. Tetrahedron.

[23] Kluba C.A., Bauman A., Valverde I.E., Vomstein S., Mindt T.L. (2012). Dual-targeting conjugates designed to improve the efficacy of radiolabeled peptides. Org. Biomol. Chem..

[24] Alberto R., Egli A., Abram U., Hegetschweiler K., Gramlich V., Schubiger A.P. (1994). Synthesis and reactivity of [NEt_4_]_2_[ReBr_3_(CO)_3_]. Formation and structural characterization of the clusters [NEt_4_][Re_3_(µ3-OH)(µ-OH)_3_(CO)_9_] and [NEt_4_][Re_2_(µ-OH)_3_(CO)_6_] by alkaline titration. J. Chem. Soc. Dalt. Trans..

[25] Vraka C., Nics L., Wagner K.H., Hacker M., Wadsak W., Mitterhauser M. (2017). LogP, a yesterday’s value?. Nucl. Med. Biol..

[26] OECD (2004). Test No. 117: Partition Coefficient (n-Octanol/Water), HPLC Method.

[27] Fischer C.A., Vomstein S., Mindt T.L. (2014). A bombesin-shepherdin radioconjugate designed for combined extra- and intracellular targeting. Pharmaceuticals.

[28] Schweinsberg C., Maes V., Brans L., Bläuenstein P., Tourwé D.A., Schubiger A.P., Schibli R., García-Garayoa E. (2008). Novel glycated [^99m^Tc(CO)_3_]-labeled bombesin analogues for improved targeting of gastrin-releasing peptide receptor-positive tumors. Bioconjug. Chem..

