# Supplementary material for: Sorbitol as a Polar Pharmacological Modifier to Enhance the Hydrophilicity of 99mTc-Tricarbonyl-Based Radiopharmaceuticals
Source: Molecules. 2020 Jun 9;25(11):2680. doi: 10.3390/molecules25112680 (PMC7321340; doi:10.3390/molecules25112680)
Supplement: Supplementary file 1 [file molecules-25-02680-s001.pdf]

Supporting Information for:

Sorbitol as a Polar Pharmacological Modifier to Enhance the Hydrophilicity of ^{99m}Tc-Tricarbonyl-Based Radiopharmaceuticals

Carolina Giammei [1,2,3,†], Theresa Balber [1,3,†], Katarina Benčurová [1,3], Jens Cardinale [1,3], Neydher Berroterán-Infante [2,3], Marie Brandt [1,3], Nedra Jouini [1,2,3], Marcus Hacker [3], Markus Mitterhauser [1,3] and Thomas L. Mindt [1,2,3,*]

[1] Ludwig Boltzmann Institute Applied Diagnostics, General Hospital of Vienna, c/o Sekretariat Nuklearmedizin, Währiger Gürtel 18-20, 1090 Vienna, Austria; carolina.giammei@lbiad.lbg.ac.at (C.G.); theresa.balber@lbiad.lbg.ac.at (T.B.); katarina.bencurova@lbiad.lbg.ac.at (K.B.); jens.cardinale@lbiad.lbg.ac.at (J.C.); marie.brandt@lbiad.lbg.ac.at (M.B.); nedra.jouini@lbiad.lbg.ac.at (N.J.); markus.mitterhauser@meduniwien.ac.at (M.M.); thomas.mindt@lbiad.lbg.ac.at (T.L.M.)

[2] Department of Inorganic Chemistry, Faculty of Chemistry, University of Vienna, Währinger Strasse 42, 1090 Vienna, Austria; neydher.berroteraninfante@meduniwien.ac.at

[3] Department of Biomedical Imaging and Image-Guided Therapy, Division of Nuclear Medicine, Medical University of Vienna, Währinger Gürtel 18-20, 1090 Vienna, Austria; marcus.hacker@meduniwien.ac.at

† Authors contributed equally to the work

* Correspondence: thomas.mindt@lbiad.lbg.ac.at

Academic editor: Fabio Zobi

Received: date; Accepted: date; Published: date

Table of Content

Characterization of compounds 2

Compound 3 2

Compound 4 3

Compound 5 4

Compound 6 5

Compound 9 6

Compound 10 8

BBN-**12** 10

HPLC 11

TLC 13

Characterization of compounds

Compound 3

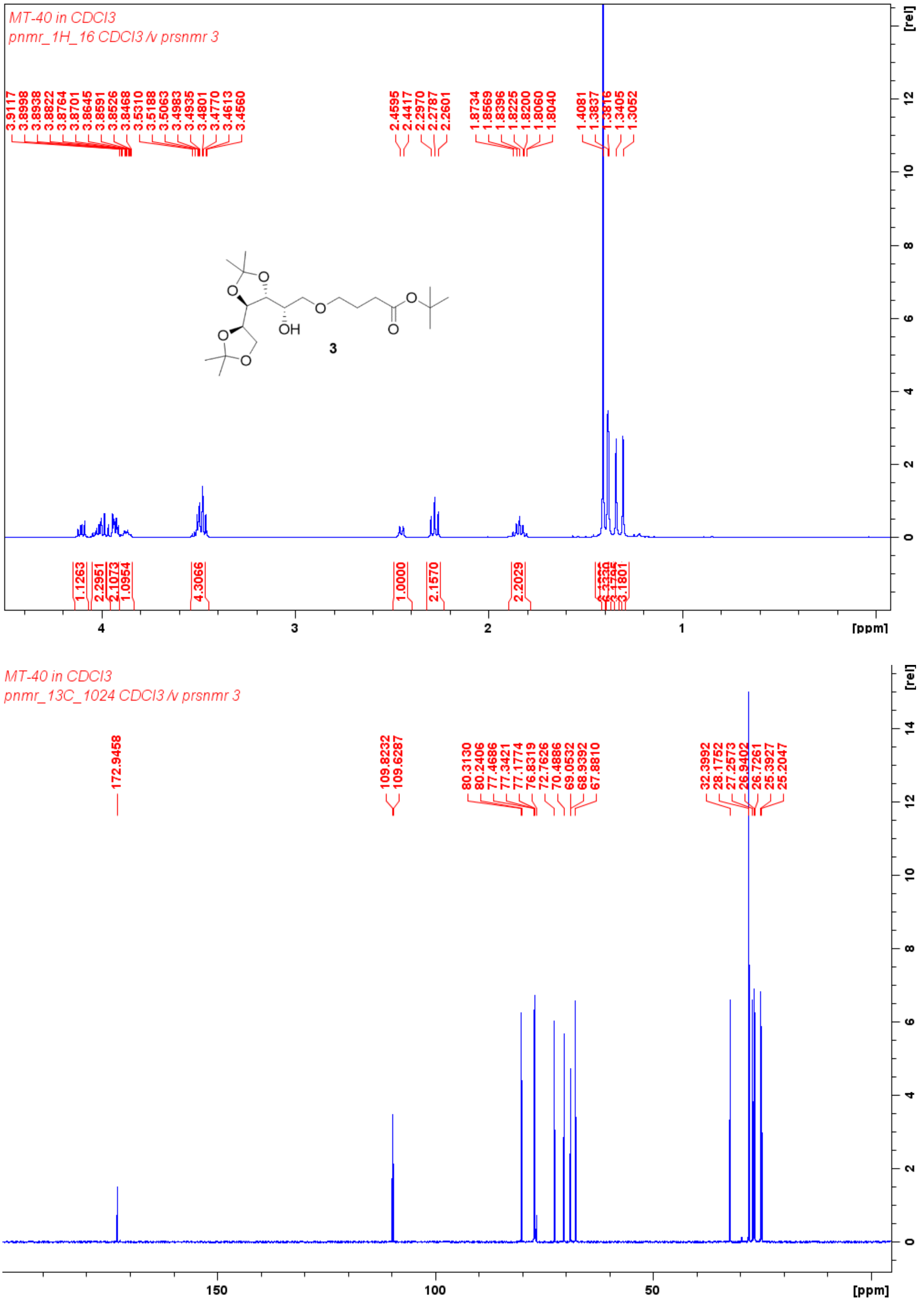

Figure S1. ^{1}H-, ^{13}C-NMR spectra of compound **3**.

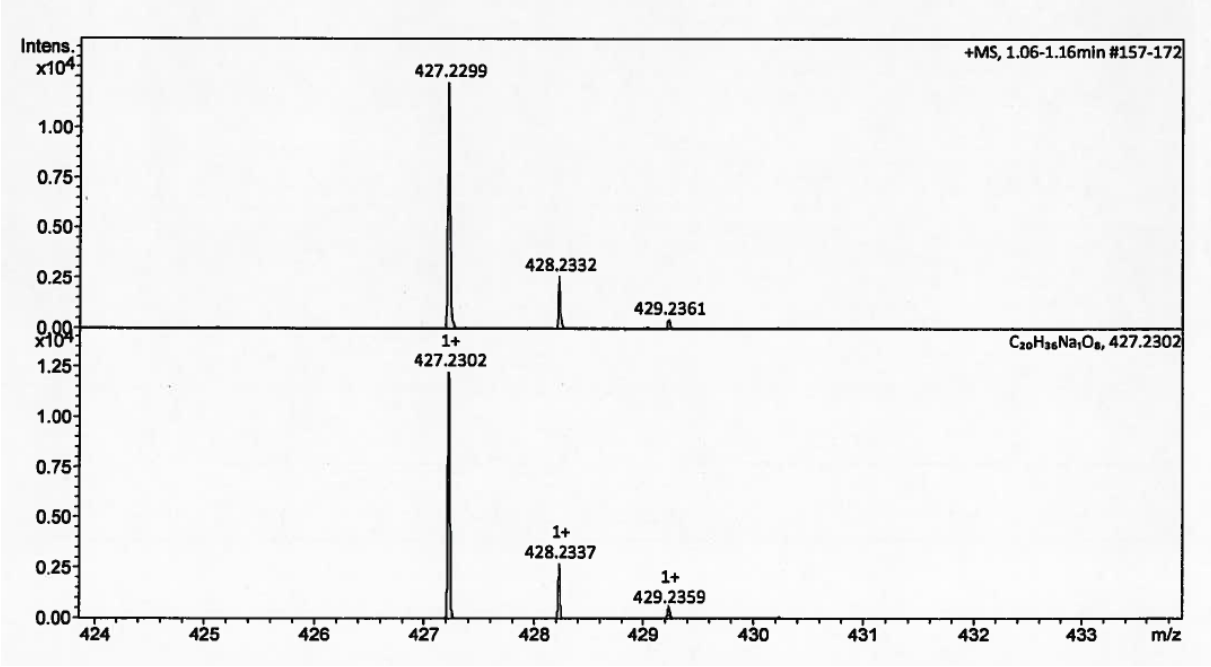

Figure S2. LR-MS data of compound **3**.

Compound 4

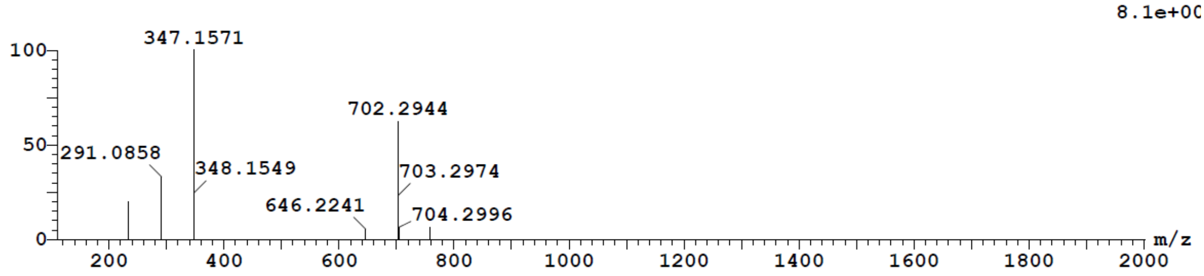

Figure S3. LR-MS data of compound **4**.

Compound 5

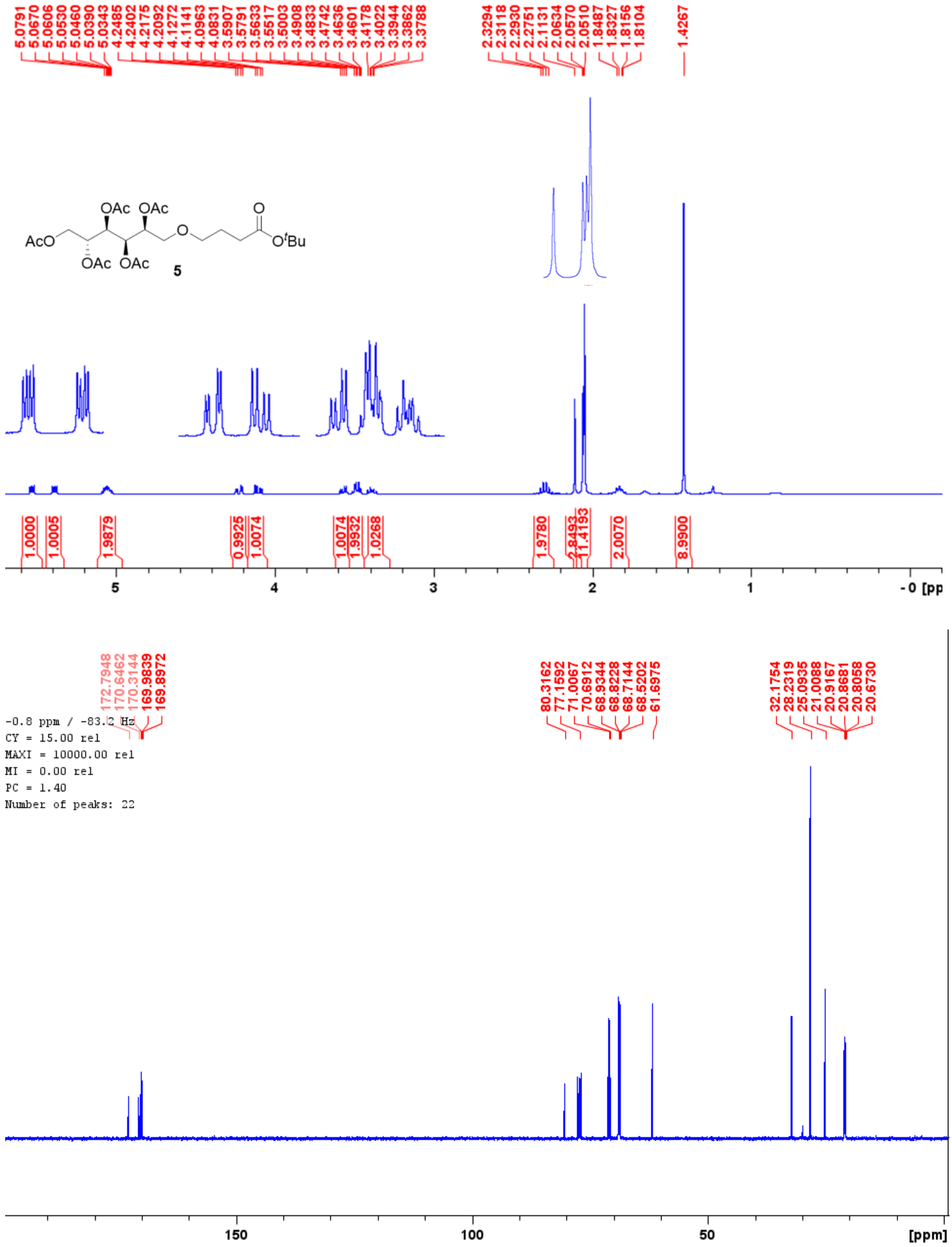

Figure S4. ^{1}H- and ^{13}C-NMR spectra of compound **5**.

Compound 6

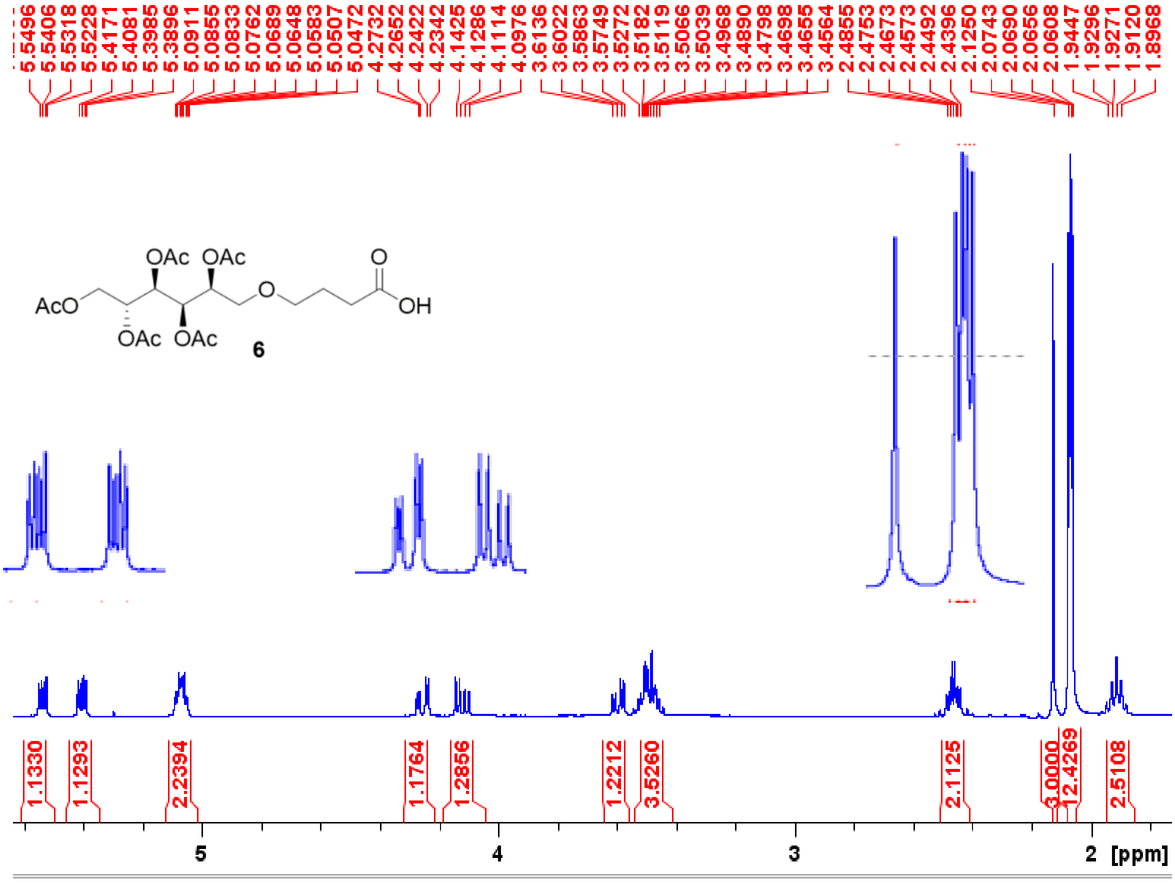

Figure S5. ^{1}H-NMR spectra of compound **6**.

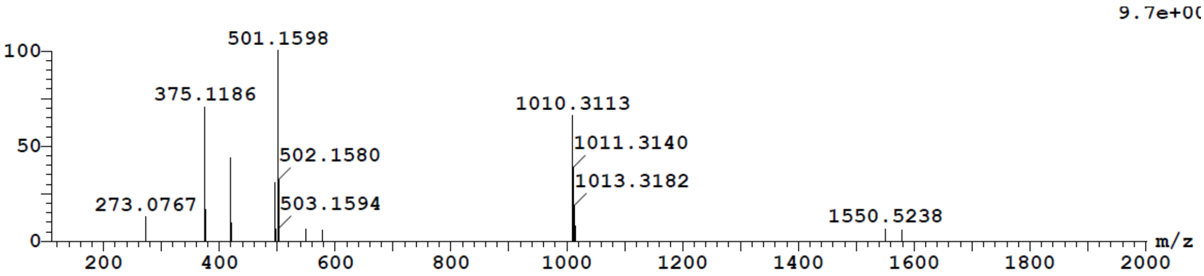

Figure S6. ^{1}H-NMR spectra of compound **6**.

Compound 9

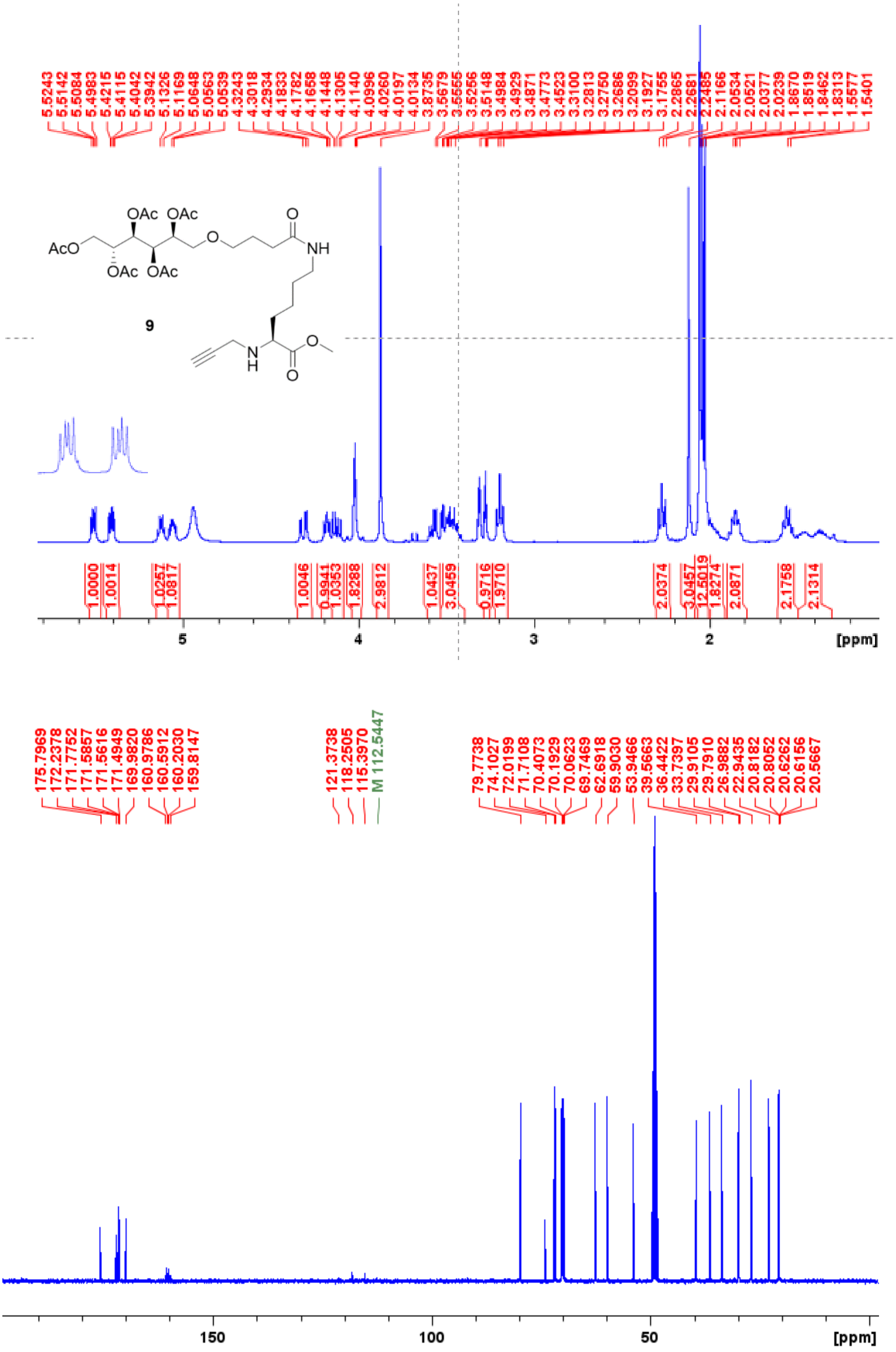

Figure S7. ^{1}H-, ^{13}C-NMR spectra of compound **9**

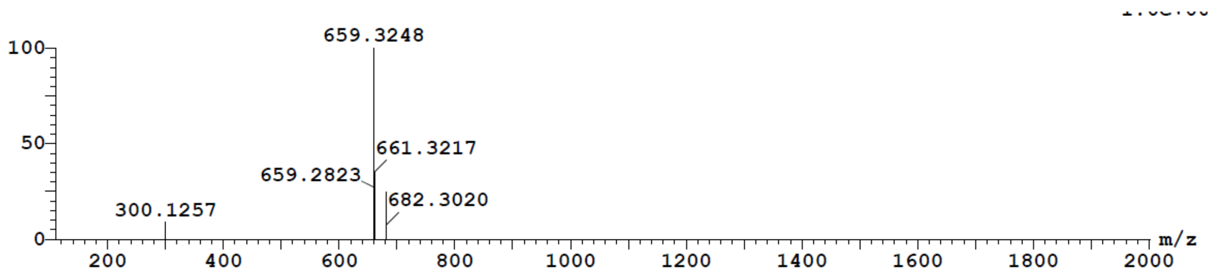

Figure S8. LR-MS data of compound **9**.

Compound 10

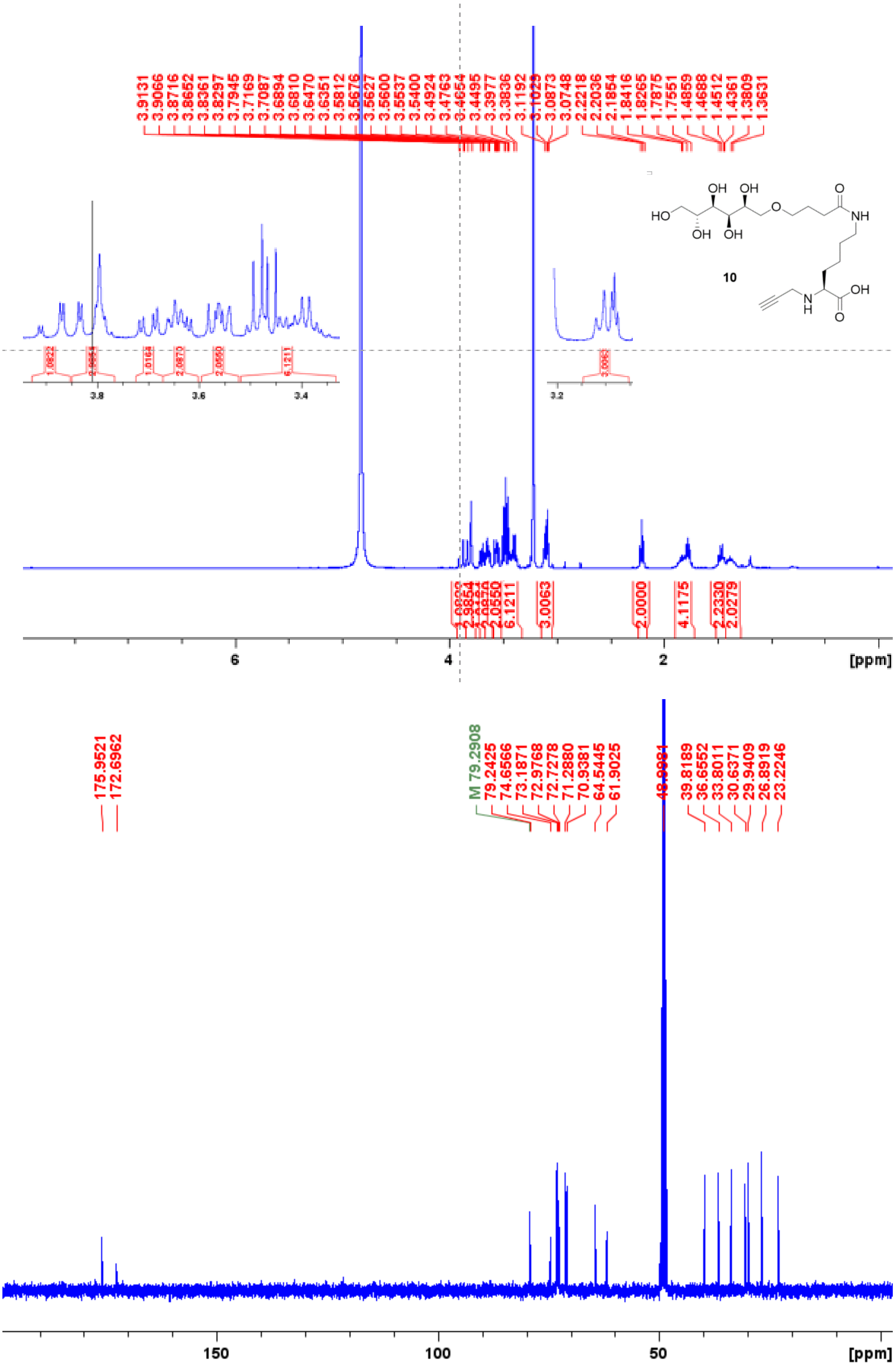

Figure S9. ^{1}H-, ^{13}C-NMR spectra of compound **10**.

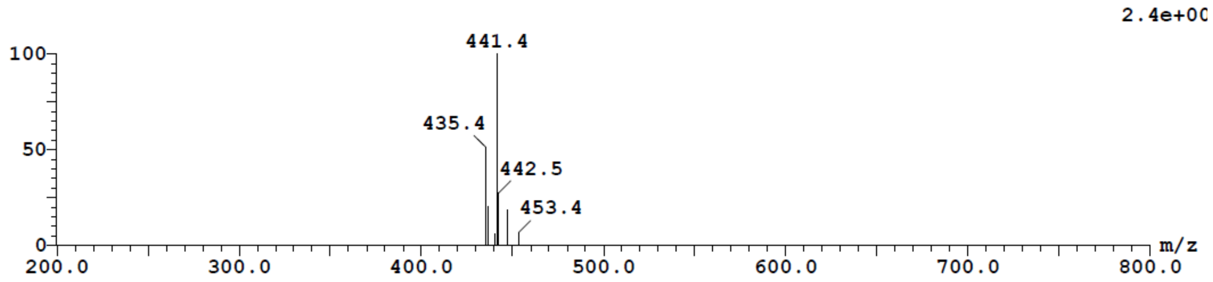

Figure S10. LR-MS data of compound **10**.

BBN-**12**

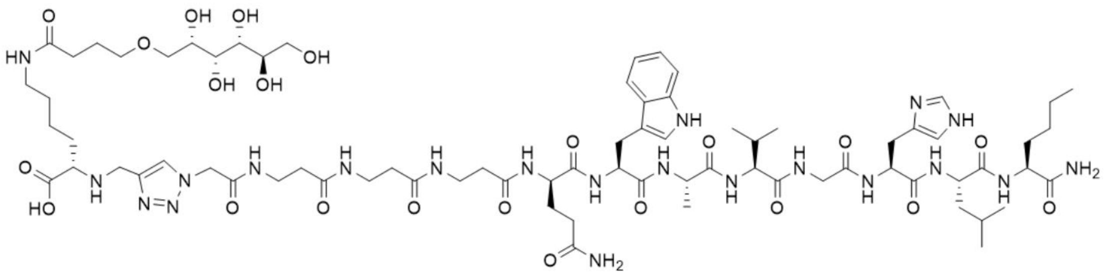

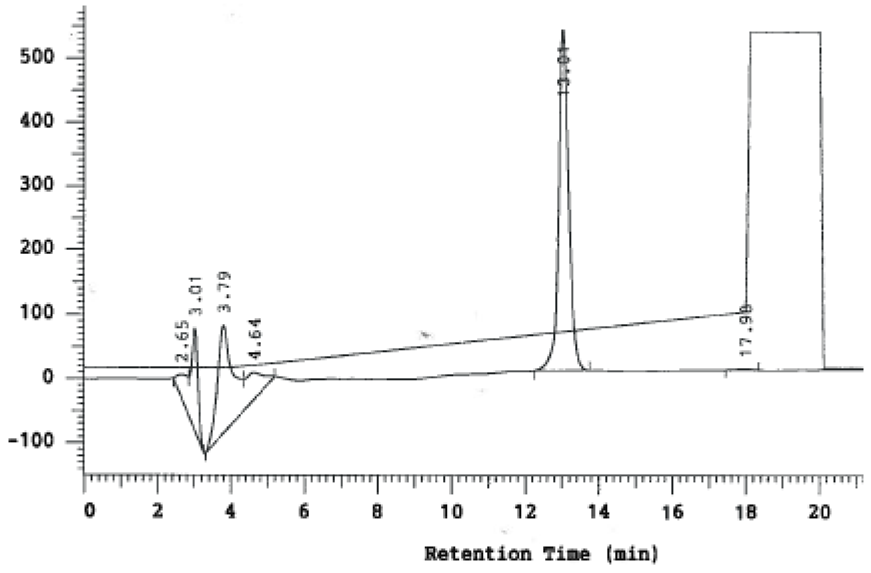

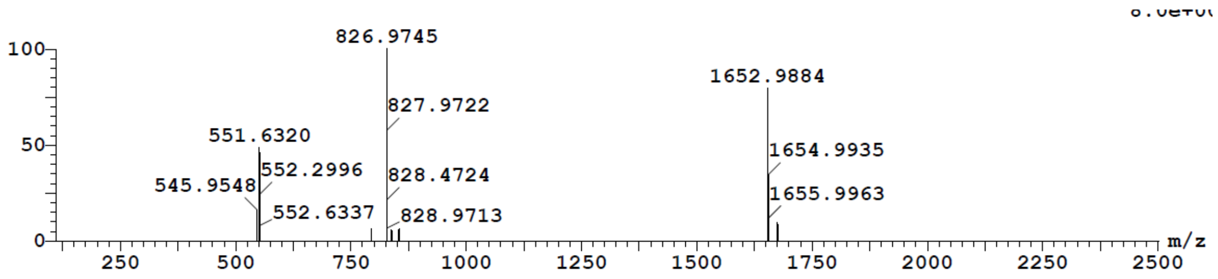

Figure S11. UV-HPLC chromatogram (λ = 215 nm, 23-35% ACN in 0.1% aq. TFA, 15 min, 1 mL/min) and LR-MS data of glycated BBN-**12**

HPLC

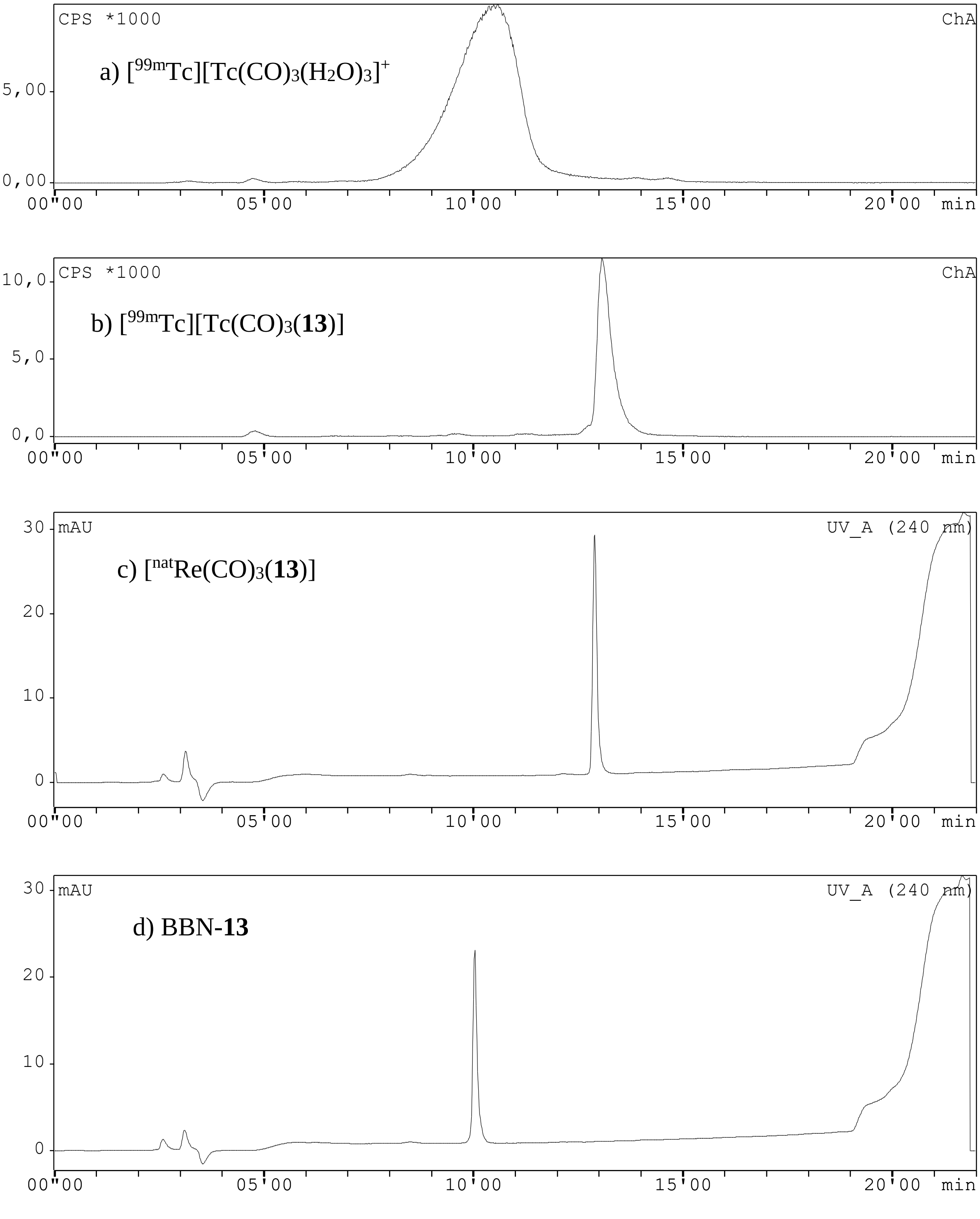

Figure S12. HPLC chromatograms (λ = 240 nm, 20-50% ACN in 0.1% TFA, 16 min, 1 mL/min) a) [^{99m}Tc][Tc(CO)$_3$(H$_2$O)$_3$]$^+$, b) [^{99m}Tc][Tc(CO)$_3$(**13**)], c) [^{nat}Re(CO)$_3$(**13**)], d) BBN-**13.** Slight differences in retention times of the ^{99m}Tc- and ^{nat}Re-compounds is due to the serial arrangement of detectors (Gamma detector behind HPLC system). The remaining difference is due to the non-isotopic reference compound.

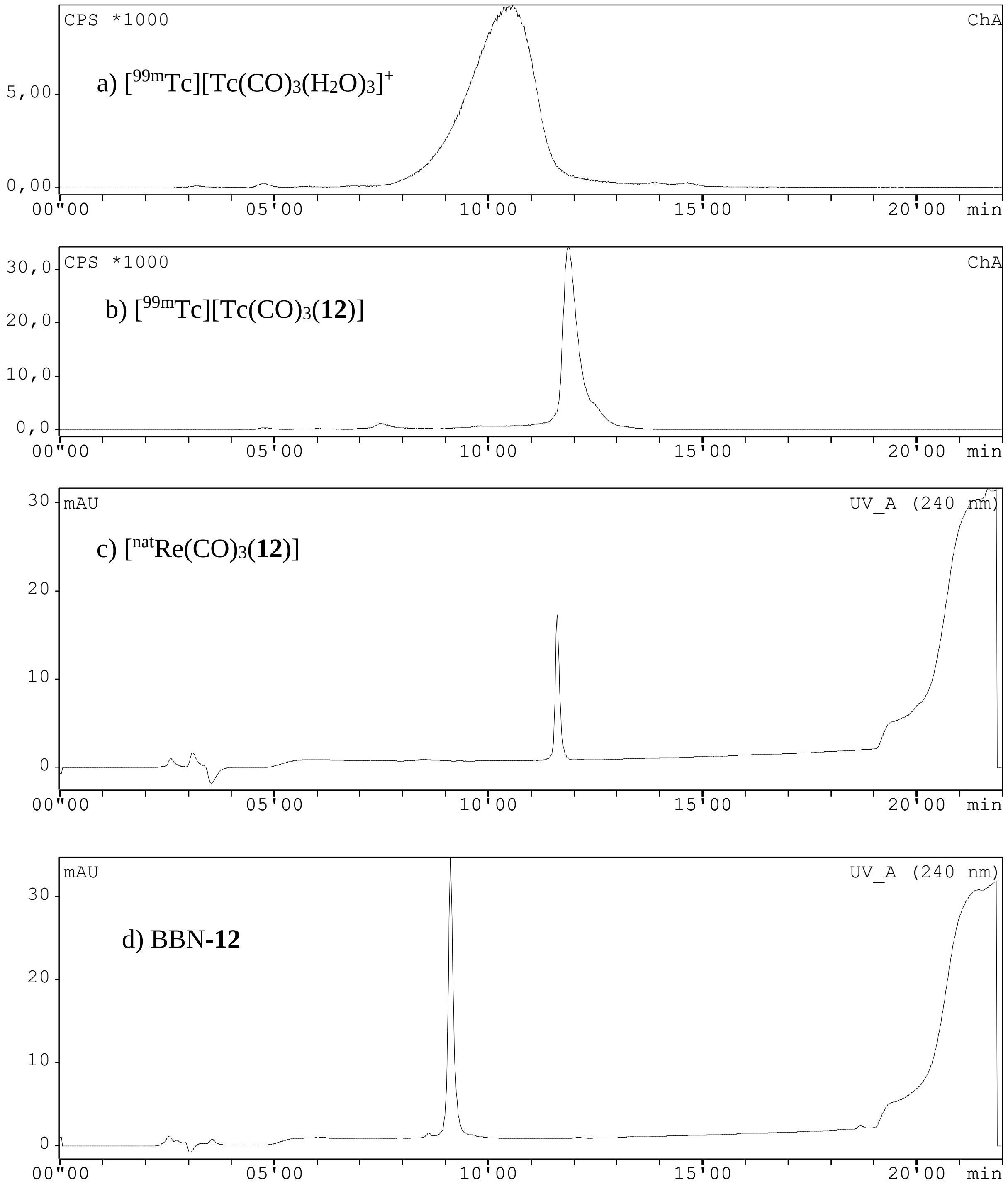

Figure S13. HPLC chromatograms (λ = 240 nm, 20-50% ACN in 0.1% TFA, 16 min, 1 mL/min) a) [^{99m}Tc][Tc(CO)$_3$(H$_2$O)$_3$]$^+$, b) [^{99m}Tc][Tc(CO)$_3$(**12**)], c) [^{nat}Re(CO)$_3$(**12**)], d) BBN-**12.** Slight differences in retention times of the ^{99m}Tc- and ^{nat}Re-compounds is due to the serial arrangement of detectors (Gamma detector behind HPLC system). The remaining difference is due to the non-isotopic reference compound.

MS

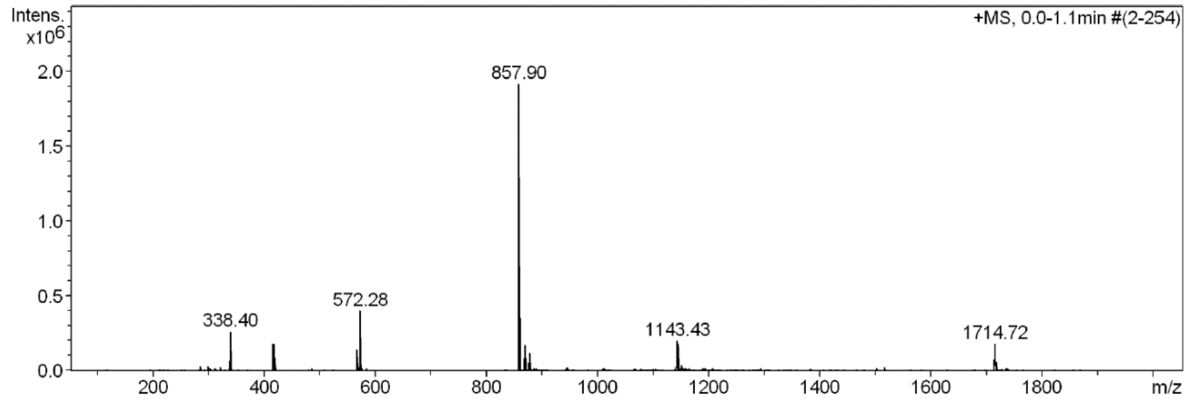

Figure S14. LR-MS data of [Re(CO)$_3$(**13**)]

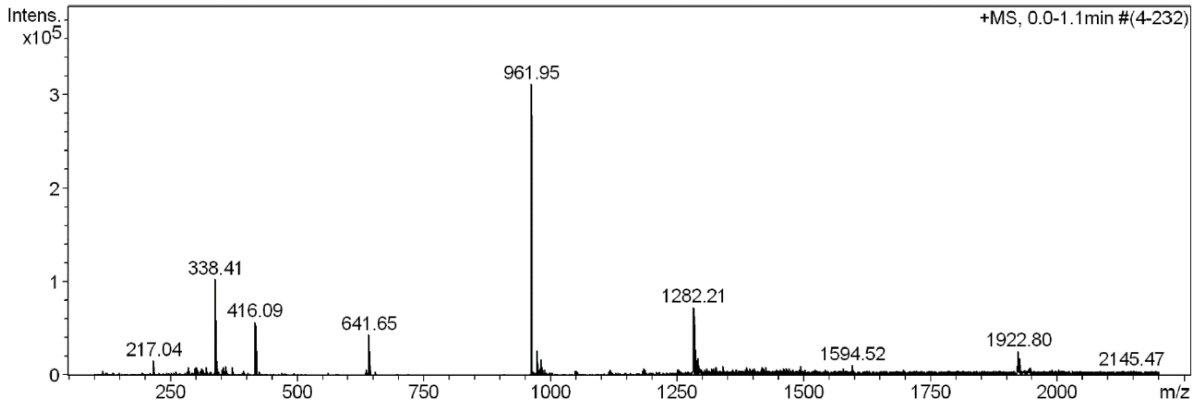

Figure S15. LR-MS data of [Re(CO)$_3$(**12**)]

TLC

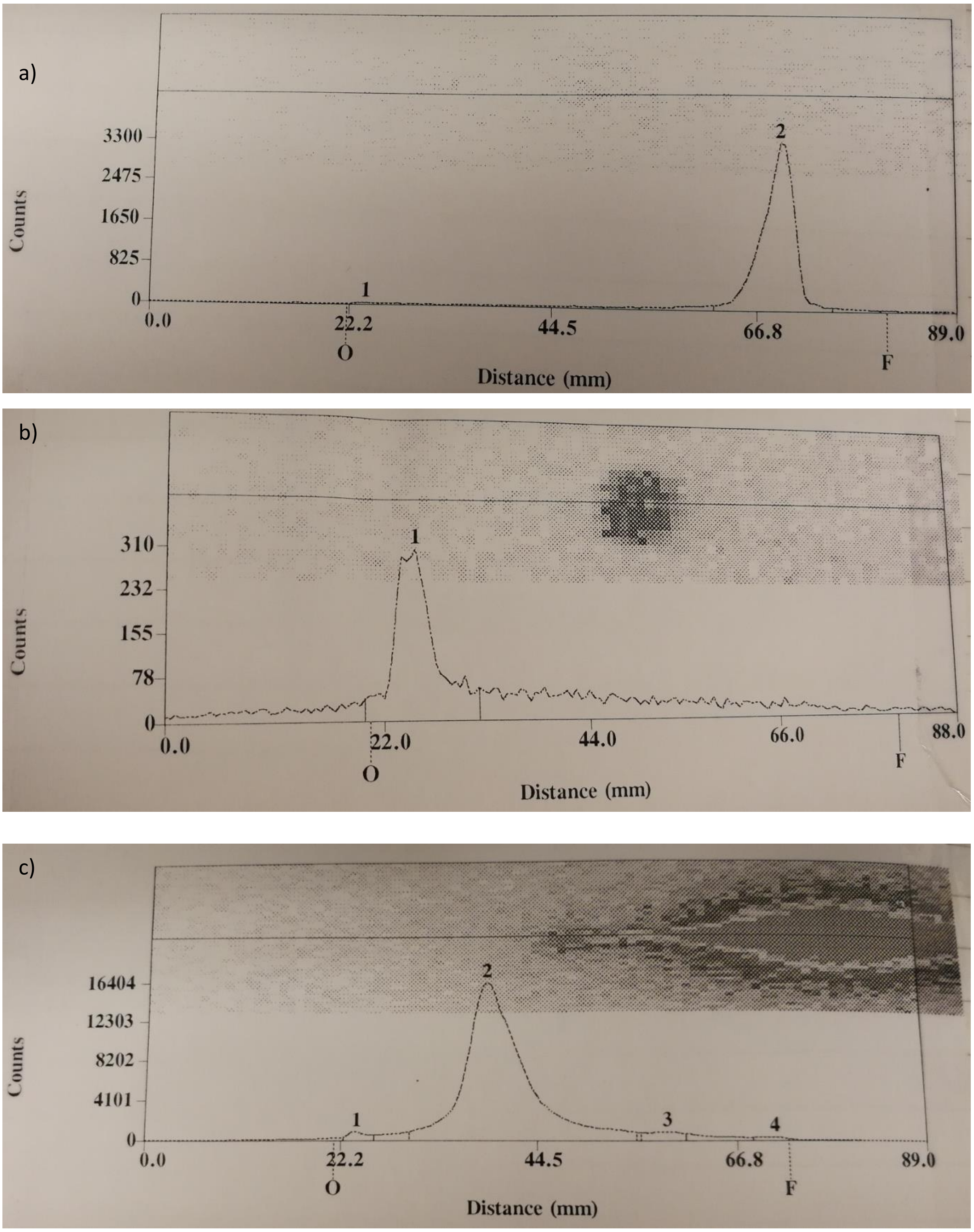

Figure S16. Scans of TLC chromatograms (mobile face 95.5:0.5 of MeOH:HCl) of a) [^{99m}Tc]TcO_4^-, b) [^{99m}Tc][$Tc(CO)_3(H_2O)_3$]$^+$, and c) [^{99m}Tc][$Tc(CO)_3$(**12**)]
